# Comparative study of heat and mass transfer of generalized MHD Oldroyd-B bio-nano fluid in a permeable medium with ramped conditions

**DOI:** 10.1038/s41598-021-02326-8

**Published:** 2021-12-06

**Authors:** Fuzhang Wang, Sadique Rehman, Jamel Bouslimi, Hammad Khaliq, Muhammad Imran Qureshi, Muhammad Kamran, Abdulaziz N. Alharbi, Hijaz Ahmad, Aamir Farooq

**Affiliations:** 1 Nanchang Institute of Technology, Nanchang, 30044 China; 2 School of Mathematics and Statistics, Xuzhou University of Technology, Xuzhou, 221018 China; 3 Department of Pure and Applied Mathematics, University of Haripur, Haripur, KPK, Pakistan; 4 Department of Physics, Faculty of Science, Taif University, P.O. Box 888, Taif, 21944 Saudi Arabia; 5 Department of Mathematics, COMSATS University Islamabad, Vehari Campus, Vehari, 61100 Pakistan; 6 Department of Mathematics, COMSATS University Islamabad, Wah Campus, Islamabad, 47040 Pakistan; 7 Department of Physics, College of Science, Taif University, P. O. Pox 11099, Taif, 21944 Saudi Arabia; 8 Department of Basic Sciences, University of Engineering Technology, Peshawar, Pakistan; 9 Section of Mathematics, International Telematic University Uninettanu, Corso Vittorio Emanuele II, 39, 00186 Roma, Italy; 10 Department of Mathematics, Abbottabad University of Science and Technology, Abbottabad, Pakistan

**Keywords:** Engineering, Mathematics and computing, Nanoscience and technology, Physics

## Abstract

This article aims to investigate the heat and mass transfer of MHD Oldroyd-B fluid with ramped conditions. The Oldroyd-B fluid is taken as a base fluid (Blood) with a suspension of gold nano-particles, to make the solution of non-Newtonian bio-magnetic nanofluid. The surface medium is taken porous. The well-known equation of Oldroyd-B nano-fluid of integer order derivative has been generalized to a non-integer order derivative. Three different types of definitions of fractional differential operators, like Caputo, Caputo-Fabrizio, Atangana-Baleanu (will be called later as $C,CF,AB$) are used to develop the resulting fractional nano-fluid model. The solution for temperature, concentration, and velocity profiles is obtained via Laplace transform and for inverse two different numerical algorithms like Zakian’s, Stehfest’s are utilized. The solutions are also shown in tabular form. To see the physical meaning of various parameters like thermal Grashof number, Radiation factor, mass Grashof number, Schmidt number, Prandtl number etc. are explained graphically and theoretically. The velocity and temperature of nanofluid decrease with increasing the value of gold nanoparticles, while increase with increasing the value of both thermal Grashof number and mass Grashof number. The Prandtl number shows opposite behavior for both temperature and velocity field. It will decelerate both the profile. Also, a comparative analysis is also presented between ours and the existing findings.

## Introduction

Non-Newtonian fluids have much more applications in science and technology than Newtonian fluids. Non-Newtonian fluids are still used in manufacturing of variety of extremely concentrated products, including fabrics, carbon, glass, and paints. Many non-Newtonian fluids are found in foods such as jams, chocolate, mayonnaise, and other condiments. Toothpaste also contains certain non-Newtonian fluids. The certain models that explain the non-Newtonian behavior of fluids are Oldroyd-B, Maxwell, Jeffry, second and third-grade, Casson^1–5^. These models are simple but each model has its own limitations. For example, the behavior of fluids with shear dangling viscosity is well supported by the power-law model, the elastic behavior does not include in the power-law model. Similarly, second and third grade show the inverse effects, they show the elasticity but viscosity for shear doesn’t depend on such models. Furthermore, these fluid models don’t convey the stress-relaxation period.

Nano-fluids are also non-Newtonian fluids, and this research focuses on nano-fluids. Choi et al.^6^ is the first who introduced the principle of nano-fluid in 1995. Such fluids are suspensions of nano-sized particles in a base fluid, such as metals, carbides, and oxides. The nano-fluids model produces very useful applications. Nanofluids contain a suspension of nanomaterials and show many interesting properties. Some of the applications involve enhanced heat transfer in electronic appliances, heavy-duty engines, industrial cooling, nuclear system cooling, heating building and reducing pollution, cooling of space defense equipment, vehicular brake fluids. In this article we are interested in Bio-magnetic fluid, because Bio-magnetic fluid dynamic (BFD) is a relatively new area in fluid mechanics investigating the fluid dynamics of biological fluids in the presence of magnetic field. The application of magnetic field on the flow of biological fluids is addressed for bioengineering and medical applications like drug targeting, cell separation, or reduction of blood flow during surgeries. The most characteristics bio-magnetic fluid is blood. Here blood is taken as a base fluid and gold nano-particles introduced in it. Das et al.^7^ pioneered the comprehensive discussion, relevance, and potential scope of nanofluids. Nano fluids had slightly higher thermal conductivity than base fluids, according to Wong and Kurma^8^. In fluid dynamics, fractional derivatives models have been discussed for viscoelastic materials, like glassy state and polymers. Recently, real life problems have been analyzed via fractional time derivative operator, like Caputo^9^, Caputo-Fabrizio^10^, and Atangana-Baleanu^11^. Fractional calculus is an emerging field which is based on various types of kernels. The main application of kernels is to provide a better description of the dynamics among complex systems, for example, collecting the memory at whole and partial domain of certain processes. The non-locality of the new kernel investigated the memory structure with alternate scales. Asifa et al.^12^ investigated the generalized MHD transport of rate-type fluid under Newtonian heating and non-uniform velocity conditions near an unbounded upright plate. Free convection flow of various MHD fluid models using fractional-order derivative having local and non-local kernel was analyzed by^13–20^. Saqib et al.^21^ deliberated the blood-gold nanofluid in a permeable medium with ramped conditions. They took Oldroyd-B fluid as a base fluid (Blood). In this they utilized the Laplace transform to acquire the solutions for temperature and velocity profiles of the model. Generalized magnetic blood flow with dust particles in cylindrical shape was studied by Saqib et al.^22^. Misra et al.^23^ investigated the bio-magnetic fluid over an extended sheet numerically using FDM. Apparent viscosity of blood in the presence of magnetic effect was addressed by Haik et al.^24^. Papadopoulos et al.^25^ was examined blood as bio-magnetic fluid in rectangular duct numerically. Hussain et al.^26^ researched the peristaltic transport of gold nanoparticles using blood as base fluid. In this, they obtained analytical solutions by the homotopy analysis method (HAM). The influence of nanosolid particle shapes, permeability material, viscous dissipative flow, Cattaneo-Christov heat flux and radiate flux are studied by Jamshed. et al.^27^. The predominant flow equations are systemized in form of PDEs. Keller-box's computational method was employed to identify the self-similar solution for transformed principles into ODEs by appropriate transformations. Saqib et al.^28^ considered the ferro-nanofluid with Brinkman type fractional model for the first time using ramped heating. LT was used to obtain the solution of the fractional CF model. The base fluid is considered as Casson fluid (blood) and CNT’s nanoparticles present inside it was illustrated by Khalid et al.^29^. They used LT to acquire the exact solution of the ordinary model. Aman et al.^30^ explained the hybrid nanoparticles in the well-known model of MHD Casson nanofluid in a permeable medium. Waqas et al.^31^ studied the magnetohydrodynamic flow of Burgers nanofluid with the interaction of nonlinear thermal radiation, activation energy, and motile micro-organisms across a stretching cylinder. The developed PDEs are transformed into a system of ODEs with the help of similarity transformations. The extracted problem is rectified numerically by using the bvp4c program in computational software MATLAB. Recently, Arshad et al.^32^ investigated bio-convective hybrid nanoparticles on a stirring needle in the presence of micro-organisms considered chemical reaction and viscous dissipation. HAM was used to acquire the analytical solution. MHD natural convection flow of nanofluid and CNT’s Oldroyd- B nanofluid in the presence of ramped conditions was illustrated by^33,34^. Entropy optimization of a system is important step of modeling^35,36^. The solution to the constructed problem is achieved by using the innovative CVFEM approach. Shah. et al.^37^ investigated the thermal effect for a mixed convection flow of Maxwell nanofluid spinning motion produced by rotating and bidirectional stretching cylinder. The modeled equations are converted to set of ODEs through group of similar variables and are then solved by using semi analytical technique HAM. Jamshed et al.^38^ studied the entropy generation in a magnetohydrodynamic flow of a Maxwell nanofluid over an infinite horizontal surface. Similarity solutions are obtained by transformation of governing PDEs to ODEs using similarity variables. Keller box method is then adopted to find the approximate solutions of reduced ODEs. The natural convection flow of different hybrid nanofluid over a radiative vertical plate and extended surfaces is discussed by^39–41^. Moured et al.^42^ studied the heat transfer and steady magneto-hydrodynamic natural convection in a fined cold wavy-walled porous enclosure with a hot elliptic inner cylinder occupied by hybrid Fe_3_O_4_-MWCNT /water nanofluid. The enclosure is also placed in a uniform magnetic field. The governing equations are verified by using the Galerkin Finite Element Method (GFEM). A single-phase mathematical model of Casson and second-grade nanofluid past over a stretching surface was analyzed by^43,44^. The Cattaneo-Christov based and unsteady Casson nanofluid over a stretching surface is investigated by^45,46^. The well-known nonlinear ODEs are solved via Keller-Box method.

The influence of a chemical reaction is determined by whether it is homogeneous or heterogeneous. The inclusion of pure water and air is impossible in nature. It's possible that any outer matter is naturally there, or that it's mixed with air or water. When an outer mass is present in air or liquids, it induces a chemical reaction. Many chemical technologies, such as the manufacture of glassware or ceramics, the production of polymers, and food processing, benefit from the study of related chemical reactions. Mazhar et al.^47^ studied the ramped wall temperature and velocity of MHD Oldroyd-B fluid of convective flow. For inverse LT, Stehfest’s algorithms are utilized. Anwar et al.^48^ extended the work of^47^, in this, they added $\left( {1 + \lambda \frac{\partial }{\partial t}} \right)$ with the coefficient of thermal expansion. Permeability is the property of porous medium which gives information how easily a fluid can pass through the porous medium. Flow via porous media is very common in nature. The concept of porous media is used in many areas of applied science and engineering, such as filtration, mechanics, petroleum engineering, geosciences, biology, and biophysics. Two important current fields of application for porous materials are energy conversion and energy storage, where porous materials are essential for supercapacitors, fuel cells, and batteries. Iftikhar et al.^49^ illustrated the concept of heat and mass transfer of MHD Oldroyd-B fluid in a permeable medium using ramped conditions. They saw the comparison between three various types of non-integer order derivatives. Mehmood et al.^50^ studied the non-Fourier heat flux model of MHD Oldroyd-B fluid of oblique stagnation flow numerically. Runge–Kutta-Fehlberg quadrature and shooting method was utilized to acquire the solution. In^51,52^, authors studied the MHD Oldroyd-B nanofluid heat and mass transmission OHAM. Electro-osmotic slip flow of generalized Oldroyd-B fluid was researched in^53,54^. Soret and Hall effects of radiating chemically MHD fluid past on a vertical plate in a rotating system with ramped temperature also concentration in a permeable medium was discussed by Seth et al.^55,56^. Narahari et al.^57^ investigated the natural convection current impact of heat and mass transmission with ramped temperature. The parabolic trough solar collector (PTSC) is widely installed in concentrated solar power technology with a temperature range of 325–700 K, deliberated by^58–61^. The Keller box numerical scheme was used to find the solution of the resulting nonlinear ODEs.

The basic purpose of this investigation is to study the bio-magnetic Oldroyd-B nanofluid with ramped conditions. However, ramped velocity and ramped temperature have attracted a little attention of the researchers examining such flows. The simultaneous use of ramped wall temperature and velocity conditions are physically important, but mathematically relations are extremely intricate and handling them analytically is sometimes troublesome. Operating ramped wall temperature and ramped wall velocity is highly significant in various subdivisions in present-day technology and science. For instance, ramped velocity is useful in evaluating the functioning of heart and blood vessels. Diagnoses of cardiovascular deceases, determining treatment and establishing prognosis involve treadmill testing and Ergometry, which operate on the basis of ramped velocity. To see the behavior of fluid flow, fractional order model is good to explain the dynamics and memory effect with respect to classical model. Three types of non-integers definitions of Caputo, Caputo-Fabrizio, and Atangana-Baleanu are used to develop the given nano-fluid model. LT and numerical Laplace inverse algorithms including Zakian’s^62^, Stehfest’s^63^ are utilized to acquire the solution of temperature, concentration, and velocity. Finally, show the impacts of physical parameters on temperature and velocity fields that are present in the problem graphically as well as theoretically.

## Model of flow problem

**Figure 1:**
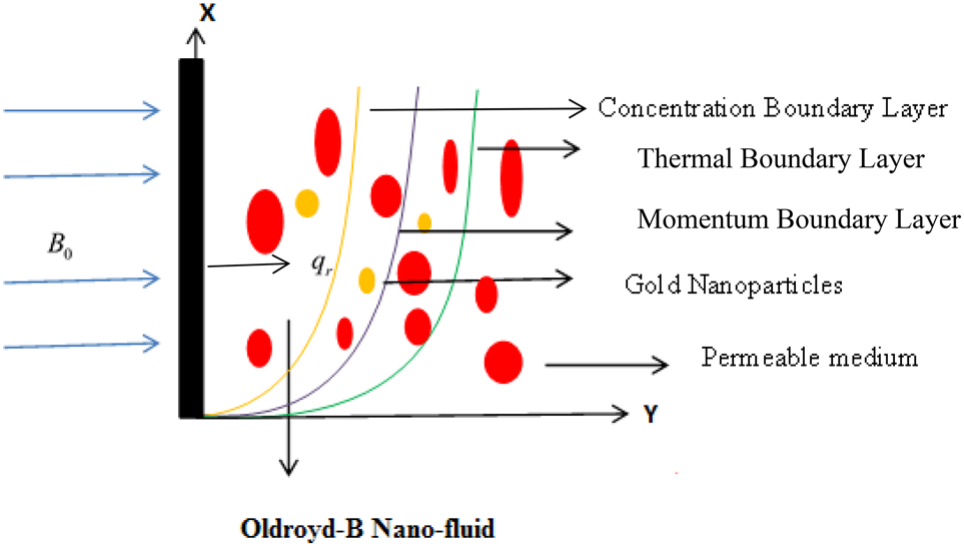
Flow geometry of the problem.

Consider the unsteady flow of Oldroyd-B nano-fluid with heat and mass transmission. The vertical plate is infinite and the medium is considered to be porous. The fluid is assumed to be electrically conducting, as a result, the external magnetic force is applied parallel to the flow direction. Both the fluid and the plate are initially at rest. After some time at $$t = 0^{ + }$$ the plate begins oscillation in its plane ($$Y = 0$$) as indicated with ramped velocity. $$Y$$ is perpendicular to the plate while plate is along X-axis. The fluid starts movement along X-direction. The geometry of the flow problem is portrayed in Fig. 1. The governing equation of the respective problem is taken from^21,49^. However, in the work of Saqib et al.^21^, the term $$1 + \overset{\lower0.5em\hbox{$\smash{\scriptscriptstyle\frown}$}}{\tilde{\lambda }}_{m} \frac{\partial }{{\partial \tilde{t}}}$$ is missing with the coefficient of thermal expansion pointing out the deficiency of their model.

1 $$\begin{aligned} \overset{\lower0.5em\hbox{$\smash{\scriptscriptstyle\frown}$}}{\tilde{\rho }}_{nf} \left( {1 + \overset{\lower0.5em\hbox{$\smash{\scriptscriptstyle\frown}$}}{\tilde{\lambda }}_{m} \frac{\partial }{{\partial \tilde{t}}}} \right)\frac{{\partial \overset{\lower0.5em\hbox{$\smash{\scriptscriptstyle\frown}$}}{\tilde{u}} \left( {\tilde{Y},\tilde{t}} \right)}}{{\partial \tilde{t}}} = & \overset{\lower0.5em\hbox{$\smash{\scriptscriptstyle\frown}$}}{\tilde{\mu }}_{nf} \left( {1 + \overset{\lower0.5em\hbox{$\smash{\scriptscriptstyle\frown}$}}{\tilde{\lambda }}_{o} \frac{\partial }{{\partial \tilde{t}}}} \right)\frac{{\partial^{2} \overset{\lower0.5em\hbox{$\smash{\scriptscriptstyle\frown}$}}{\tilde{u}} \left( {\tilde{Y},\tilde{t}} \right)}}{{\partial \tilde{Y}^{2} }} - \overset{\lower0.5em\hbox{$\smash{\scriptscriptstyle\frown}$}}{\tilde{\sigma }}_{nf} B_{0}^{2} \left( {1 + \overset{\lower0.5em\hbox{$\smash{\scriptscriptstyle\frown}$}}{\tilde{\lambda }}_{m} \frac{\partial }{{\partial \tilde{t}}}} \right)\overset{\lower0.5em\hbox{$\smash{\scriptscriptstyle\frown}$}}{\tilde{u}} \left( {\tilde{Y},\tilde{t}} \right) \\ & \; - \frac{{\overset{\lower0.5em\hbox{$\smash{\scriptscriptstyle\frown}$}}{\tilde{\mu }}_{nf} \overset{\lower0.5em\hbox{$\smash{\scriptscriptstyle\frown}$}}{\tilde{\phi }} }}{k}\left( {1 + \overset{\lower0.5em\hbox{$\smash{\scriptscriptstyle\frown}$}}{\tilde{\lambda }}_{o} \frac{\partial }{{\partial \tilde{t}}}} \right)\overset{\lower0.5em\hbox{$\smash{\scriptscriptstyle\frown}$}}{\tilde{u}} \left( {\tilde{Y},\tilde{t}} \right) + \overset{\lower0.5em\hbox{$\smash{\scriptscriptstyle\frown}$}}{g} \left( {\overset{\lower0.5em\hbox{$\smash{\scriptscriptstyle\frown}$}}{\tilde{\rho }} \overset{\lower0.5em\hbox{$\smash{\scriptscriptstyle\frown}$}}{\beta }_{T} } \right)_{nf} \left( {1 + \overset{\lower0.5em\hbox{$\smash{\scriptscriptstyle\frown}$}}{\tilde{\lambda }}_{m} \frac{\partial }{{\partial \tilde{t}}}} \right)\left( {\overset{\lower0.5em\hbox{$\smash{\scriptscriptstyle\frown}$}}{\tilde{T}} - \overset{\lower0.5em\hbox{$\smash{\scriptscriptstyle\frown}$}}{\tilde{T}}_{\infty } } \right) \\ & \; + \overset{\lower0.5em\hbox{$\smash{\scriptscriptstyle\frown}$}}{g} \left( {\overset{\lower0.5em\hbox{$\smash{\scriptscriptstyle\frown}$}}{\tilde{\rho }} \overset{\lower0.5em\hbox{$\smash{\scriptscriptstyle\frown}$}}{\beta }_{C} } \right)_{nf} \left( {1 + \overset{\lower0.5em\hbox{$\smash{\scriptscriptstyle\frown}$}}{\tilde{\lambda }}_{m} \frac{\partial }{{\partial \tilde{t}}}} \right)\left( {\overset{\lower0.5em\hbox{$\smash{\scriptscriptstyle\frown}$}}{\tilde{C}} - \overset{\lower0.5em\hbox{$\smash{\scriptscriptstyle\frown}$}}{\tilde{C}}_{\infty } } \right), \\ \end{aligned}$$

2 $$\left( {\overset{\lower0.5em\hbox{$\smash{\scriptscriptstyle\frown}$}}{\tilde{\rho }} \overset{\lower0.5em\hbox{$\smash{\scriptscriptstyle\frown}$}}{c}_{p} } \right)_{nf} \frac{{\partial \overset{\lower0.5em\hbox{$\smash{\scriptscriptstyle\frown}$}}{\tilde{T}} \left( {\tilde{Y},\tilde{t}} \right)}}{{\partial \tilde{t}}} = k_{nf} \frac{{\partial^{2} \overset{\lower0.5em\hbox{$\smash{\scriptscriptstyle\frown}$}}{\tilde{T}} \left( {\tilde{Y},\tilde{t}} \right)}}{{\partial \tilde{Y}^{2} }} - \frac{{\partial \tilde{q}_{r} }}{{\partial \tilde{Y}}},$$

3 $$\frac{{\partial \overset{\lower0.5em\hbox{$\smash{\scriptscriptstyle\frown}$}}{\tilde{C}} \left( {\tilde{Y},\tilde{t}} \right)}}{{\partial \tilde{t}}} = \overset{\lower0.5em\hbox{$\smash{\scriptscriptstyle\smile}$}}{D}_{nf} \frac{{\partial^{2} \overset{\lower0.5em\hbox{$\smash{\scriptscriptstyle\frown}$}}{\tilde{C}} \left( {\tilde{Y},\tilde{t}} \right)}}{{\partial \tilde{Y}^{2} }},$$

Using Rosseland approximations^55^ and accepting the small temperature variations among the temperature $T_{\infty }$ of the free stream and the fluid’s temperature $T,$ expanding the Taylor theorem on $T^{4}$ about $T_{\infty }$ and ignoring the 2nd and higher-order, we obtain

4 $$\tilde{q}_{r} = - \frac{{4\overline{\beta }^{*} }}{{3\overline{\delta }^{*} }}\frac{{\partial \overset{\lower0.5em\hbox{$\smash{\scriptscriptstyle\frown}$}}{\tilde{T}}^{4} \left( {\tilde{Y},\tilde{t}} \right)}}{{\partial \tilde{Y}}},$$

And

5 $$\overset{\lower0.5em\hbox{$\smash{\scriptscriptstyle\frown}$}}{\tilde{T}}^{4} \cong 4\overset{\lower0.5em\hbox{$\smash{\scriptscriptstyle\frown}$}}{\tilde{T}}_{\infty }^{3} \overset{\lower0.5em\hbox{$\smash{\scriptscriptstyle\frown}$}}{\tilde{T}} - 3\overset{\lower0.5em\hbox{$\smash{\scriptscriptstyle\frown}$}}{\tilde{T}}_{\infty }^{4} ,$$

where $\tilde{\beta }^{*} ,\tilde{\delta }^{*}$ are respectively Stefan-Boltzmann constants and the mean absorption coefficient. Introducing Eq. (4) in (2), we get

6 $$\left( {\overset{\lower0.5em\hbox{$\smash{\scriptscriptstyle\frown}$}}{\tilde{\rho }} c_{p} } \right)_{nf} \frac{{\partial \overset{\lower0.5em\hbox{$\smash{\scriptscriptstyle\frown}$}}{\tilde{T}} \left( {\tilde{Y},\tilde{t}} \right)}}{{\partial \tilde{t}}} = k_{nf} \frac{{\partial^{2} \overset{\lower0.5em\hbox{$\smash{\scriptscriptstyle\frown}$}}{\tilde{T}} \left( {\tilde{Y},\tilde{t}} \right)}}{{\partial \tilde{Y}^{2} }} + \frac{{16\overline{\beta }^{*} \overset{\lower0.5em\hbox{$\smash{\scriptscriptstyle\frown}$}}{\tilde{T}}_{\infty }^{3} }}{{3\overline{\delta }^{*} }}\frac{{\partial^{2} \overset{\lower0.5em\hbox{$\smash{\scriptscriptstyle\frown}$}}{\tilde{T}} \left( {\tilde{Y},\tilde{t}} \right)}}{{\partial \tilde{Y}^{2} }},$$

The appropriate ICs and BCs are as follow^21,55^

7 $$\begin{gathered} \overset{\lower0.5em\hbox{$\smash{\scriptscriptstyle\frown}$}}{\tilde{u}} \left( {\tilde{Y},0} \right) = 0, \, \overset{\lower0.5em\hbox{$\smash{\scriptscriptstyle\frown}$}}{\tilde{T}} \left( {\tilde{Y},0} \right) = \overset{\lower0.5em\hbox{$\smash{\scriptscriptstyle\frown}$}}{\tilde{T}}_{\infty } , \, \overset{\lower0.5em\hbox{$\smash{\scriptscriptstyle\frown}$}}{\tilde{C}} \left( {\tilde{Y},0} \right) = \overset{\lower0.5em\hbox{$\smash{\scriptscriptstyle\frown}$}}{\tilde{C}}_{\infty } , \hfill \\ \overset{\lower0.5em\hbox{$\smash{\scriptscriptstyle\frown}$}}{\tilde{u}} \left( {0,\tilde{t}} \right) = \left\{ \begin{gathered} \overset{\lower0.5em\hbox{$\smash{\scriptscriptstyle\frown}$}}{\tilde{u}}_{0} \frac{{\tilde{t}}}{{\tilde{t}_{0} }} \, 0 < \tilde{t} < \tilde{t}_{0} \hfill \\ \overset{\lower0.5em\hbox{$\smash{\scriptscriptstyle\frown}$}}{\tilde{u}}_{0} \, \tilde{t}_{0} < \tilde{t} < \infty , \hfill \\ \end{gathered} \right. \, \overset{\lower0.5em\hbox{$\smash{\scriptscriptstyle\frown}$}}{\tilde{T}} \left( {0,\tilde{t}} \right) = \left\{ \begin{gathered} \overset{\lower0.5em\hbox{$\smash{\scriptscriptstyle\frown}$}}{\tilde{T}}_{\infty } + \left( {\overset{\lower0.5em\hbox{$\smash{\scriptscriptstyle\frown}$}}{\tilde{T}}_{W} - \overset{\lower0.5em\hbox{$\smash{\scriptscriptstyle\frown}$}}{\tilde{T}}_{\infty } } \right)\frac{{\tilde{t}}}{{\tilde{t}_{0} }} \, 0 < \tilde{t} < \tilde{t}_{0} \hfill \\ \overset{\lower0.5em\hbox{$\smash{\scriptscriptstyle\frown}$}}{\tilde{T}}_{W} \, \tilde{t}_{0} < \tilde{t} < \infty , \hfill \\ \end{gathered} \right. \hfill \\ \overset{\lower0.5em\hbox{$\smash{\scriptscriptstyle\frown}$}}{\tilde{C}} \left( {0,\tilde{t}} \right) = \left\{ \begin{gathered} \overset{\lower0.5em\hbox{$\smash{\scriptscriptstyle\frown}$}}{\tilde{C}}_{\infty } + \left( {\overset{\lower0.5em\hbox{$\smash{\scriptscriptstyle\frown}$}}{\tilde{C}}_{W} - \overset{\lower0.5em\hbox{$\smash{\scriptscriptstyle\frown}$}}{\tilde{C}}_{\infty } } \right)\frac{{\tilde{t}}}{{\tilde{t}_{0} }} \, 0 < \tilde{t} < \tilde{t}_{0} \hfill \\ \overset{\lower0.5em\hbox{$\smash{\scriptscriptstyle\frown}$}}{\tilde{C}}_{W} \, \tilde{t}_{0} < \tilde{t} < \infty , \hfill \\ \end{gathered} \right. \hfill \\ \overset{\lower0.5em\hbox{$\smash{\scriptscriptstyle\frown}$}}{\tilde{u}} \left( {\tilde{Y},\tilde{t}} \right) = 0, \, \overset{\lower0.5em\hbox{$\smash{\scriptscriptstyle\frown}$}}{\tilde{T}} \left( {\tilde{Y},\tilde{t}} \right) = \overset{\lower0.5em\hbox{$\smash{\scriptscriptstyle\frown}$}}{\tilde{T}}_{\infty } , \, \overset{\lower0.5em\hbox{$\smash{\scriptscriptstyle\frown}$}}{\tilde{C}} \left( {\tilde{Y},\tilde{t}} \right) = \overset{\lower0.5em\hbox{$\smash{\scriptscriptstyle\frown}$}}{\tilde{C}}_{\infty } ,{\text{ as }}\tilde{Y} \to \infty . \hfill \\ \end{gathered}$$

The thermophysical properties of nano-fluid are given as^21^

8 $$\left. \begin{gathered} \overset{\lower0.5em\hbox{$\smash{\scriptscriptstyle\frown}$}}{\tilde{\rho }}_{nf} = \left( {1 - \ddot{\varphi }} \right)\overset{\lower0.5em\hbox{$\smash{\scriptscriptstyle\frown}$}}{\tilde{\rho }}_{f} + \ddot{\varphi }\overset{\lower0.5em\hbox{$\smash{\scriptscriptstyle\frown}$}}{\tilde{\rho }}_{s} ,\overset{\lower0.5em\hbox{$\smash{\scriptscriptstyle\frown}$}}{\tilde{\mu }}_{nf} = \frac{{\overset{\lower0.5em\hbox{$\smash{\scriptscriptstyle\frown}$}}{\tilde{\mu }}_{f} }}{{\left( {1 - \ddot{\varphi }} \right)^{2.5} }},\frac{{\overset{\lower0.5em\hbox{$\smash{\scriptscriptstyle\frown}$}}{\tilde{\sigma }}_{nf} }}{{\overset{\lower0.5em\hbox{$\smash{\scriptscriptstyle\frown}$}}{\tilde{\sigma }}_{f} }} = 1 + \frac{{3\left( {\frac{{\overset{\lower0.5em\hbox{$\smash{\scriptscriptstyle\frown}$}}{\tilde{\sigma }}_{s} }}{{\overset{\lower0.5em\hbox{$\smash{\scriptscriptstyle\frown}$}}{\tilde{\sigma }}_{f} }} - 1} \right)\ddot{\varphi }}}{{\left( {\frac{{\overset{\lower0.5em\hbox{$\smash{\scriptscriptstyle\frown}$}}{\tilde{\sigma }}_{s} }}{{\overset{\lower0.5em\hbox{$\smash{\scriptscriptstyle\frown}$}}{\tilde{\sigma }}_{f} }} + 2} \right) - \left( {\frac{{\overset{\lower0.5em\hbox{$\smash{\scriptscriptstyle\frown}$}}{\tilde{\sigma }}_{s} }}{{\overset{\lower0.5em\hbox{$\smash{\scriptscriptstyle\frown}$}}{\tilde{\sigma }}_{f} }} - 1} \right)\ddot{\varphi }}}, \hfill \\ \left( {\overset{\lower0.5em\hbox{$\smash{\scriptscriptstyle\frown}$}}{\tilde{\rho }} \overset{\lower0.5em\hbox{$\smash{\scriptscriptstyle\frown}$}}{\beta }_{T} } \right)_{nf} = \left( {1 - \ddot{\varphi }} \right)\left( {\overset{\lower0.5em\hbox{$\smash{\scriptscriptstyle\frown}$}}{\tilde{\rho }} \overset{\lower0.5em\hbox{$\smash{\scriptscriptstyle\frown}$}}{\beta }_{T} } \right)_{f} + \ddot{\varphi }\left( {\overset{\lower0.5em\hbox{$\smash{\scriptscriptstyle\frown}$}}{\tilde{\rho }} \overset{\lower0.5em\hbox{$\smash{\scriptscriptstyle\frown}$}}{\beta }_{T} } \right)_{s} ,\left( {\overset{\lower0.5em\hbox{$\smash{\scriptscriptstyle\frown}$}}{\tilde{\rho }} \overset{\lower0.5em\hbox{$\smash{\scriptscriptstyle\frown}$}}{\beta }_{C} } \right)_{nf} = \left( {1 - \ddot{\varphi }} \right)\left( {\overset{\lower0.5em\hbox{$\smash{\scriptscriptstyle\frown}$}}{\tilde{\rho }} \overset{\lower0.5em\hbox{$\smash{\scriptscriptstyle\frown}$}}{\beta }_{C} } \right)_{f} + \ddot{\varphi }\left( {\overset{\lower0.5em\hbox{$\smash{\scriptscriptstyle\frown}$}}{\tilde{\rho }} \overset{\lower0.5em\hbox{$\smash{\scriptscriptstyle\frown}$}}{\beta }_{C} } \right)_{s} , \hfill \\ \left( {\overset{\lower0.5em\hbox{$\smash{\scriptscriptstyle\frown}$}}{\tilde{\rho }} c_{p} } \right)_{nf} = \left( {1 - \ddot{\varphi }} \right)\left( {\overset{\lower0.5em\hbox{$\smash{\scriptscriptstyle\frown}$}}{\tilde{\rho }} c_{p} } \right)_{f} + \ddot{\varphi }\left( {\overset{\lower0.5em\hbox{$\smash{\scriptscriptstyle\frown}$}}{\tilde{\rho }} c_{p} } \right)_{s} ,\frac{{\overset{\lower0.5em\hbox{$\smash{\scriptscriptstyle\frown}$}}{\tilde{k}}_{nf} }}{{\overset{\lower0.5em\hbox{$\smash{\scriptscriptstyle\frown}$}}{\tilde{k}}_{f} }} = \frac{{\overset{\lower0.5em\hbox{$\smash{\scriptscriptstyle\frown}$}}{\tilde{k}}_{s} + 2\overset{\lower0.5em\hbox{$\smash{\scriptscriptstyle\frown}$}}{\tilde{k}}_{f} + 2\ddot{\varphi }\left( {\overset{\lower0.5em\hbox{$\smash{\scriptscriptstyle\frown}$}}{\tilde{k}}_{s} - \overset{\lower0.5em\hbox{$\smash{\scriptscriptstyle\frown}$}}{\tilde{k}}_{f} } \right)}}{{\overset{\lower0.5em\hbox{$\smash{\scriptscriptstyle\frown}$}}{\tilde{k}}_{s} + \overset{\lower0.5em\hbox{$\smash{\scriptscriptstyle\frown}$}}{\tilde{k}}_{f} - \ddot{\varphi }\left( {\overset{\lower0.5em\hbox{$\smash{\scriptscriptstyle\frown}$}}{\tilde{k}}_{s} - \overset{\lower0.5em\hbox{$\smash{\scriptscriptstyle\frown}$}}{\tilde{k}}_{f} } \right)}}. \hfill \\ \end{gathered} \right\}$$

where $nf,f{\text{ and }}s$ respectively represent nanofluid, base fluid and solid nano-particles. Blood is taken as a base non-Newtonian fluid, and gold nanoparticles are put on it to make nanofluid. The thermophysical estimation of blood and gold nanoparticles is presented in Table 1.

**Table 1:** shows the numerical estimation of base fluid and solid nanoparticles^21^.

Material	Base fluid (blood)	Gold nanoparticles
$$\overset{\lower0.5em\hbox{$\smash{\scriptscriptstyle\frown}$}}{\tilde{\rho }} \left( {{\raise0.7ex\hbox{${\overline{k}g}$} \!\mathord{\left/ {\vphantom {{\overline{k}g} {\overline{m}^{3} }}}\right.\kern-\nulldelimiterspace} \!\lower0.7ex\hbox{${\overline{m}^{3} }$}}} \right)$$	1053	1250
$$c_{p} \left( {{\raise0.7ex\hbox{${\ddot{J}}$} \!\mathord{\left/ {\vphantom {{\ddot{J}} {\overline{k}g}}}\right.\kern-\nulldelimiterspace} \!\lower0.7ex\hbox{${\overline{k}g}$}}\hat{K}} \right)$$	3594	129
$$\overset{\lower0.5em\hbox{$\smash{\scriptscriptstyle\frown}$}}{\tilde{k}} \left( {{{\overline{W}} \mathord{\left/ {\vphantom {{\overline{W}} {\overline{m}\hat{K}}}} \right. \kern-\nulldelimiterspace} {\overline{m}\hat{K}}}} \right)$$	0.492	318
$$\overset{\lower0.5em\hbox{$\smash{\scriptscriptstyle\frown}$}}{\beta }_{T} \times 10^{ - 5} \left( {\hat{K}^{ - 1} } \right)$$	0.8	1.41
$$\overset{\lower0.5em\hbox{$\smash{\scriptscriptstyle\frown}$}}{\tilde{\sigma }} \left( {{{\hat{S}} \mathord{\left/ {\vphantom {{\hat{S}} {\overline{m}}}} \right. \kern-\nulldelimiterspace} {\overline{m}}}} \right)$$	0.18	$$4.1 \times 10^{ - 7}$$

## Blood-gold nanofluid fractional derivative model

Consider the following dimensionless parameters

9 $$\left. {\overset{\lower0.5em\hbox{$\smash{\scriptscriptstyle\smile}$}}{U}^{ * } = \frac{{\overset{\lower0.5em\hbox{$\smash{\scriptscriptstyle\frown}$}}{\tilde{u}} }}{{\overset{\lower0.5em\hbox{$\smash{\scriptscriptstyle\frown}$}}{\tilde{u}}_{0} }},\overline{\tilde{Y}} = \frac{{\overset{\lower0.5em\hbox{$\smash{\scriptscriptstyle\frown}$}}{\tilde{u}}_{0} }}{{\ddot{\upsilon }_{f} }},\overline{\tilde{t}} = \frac{{\tilde{t}}}{{\tilde{t}_{0} }},\tilde{t}_{0} = \frac{{\ddot{\upsilon }_{f} }}{{\overset{\lower0.5em\hbox{$\smash{\scriptscriptstyle\frown}$}}{\tilde{u}}_{0}^{2} }},\overset{\lower0.5em\hbox{$\smash{\scriptscriptstyle\frown}$}}{\Phi } = \frac{{\overset{\lower0.5em\hbox{$\smash{\scriptscriptstyle\frown}$}}{\tilde{T}} - \overset{\lower0.5em\hbox{$\smash{\scriptscriptstyle\frown}$}}{\tilde{T}}_{\infty } }}{{\overset{\lower0.5em\hbox{$\smash{\scriptscriptstyle\frown}$}}{\tilde{T}}_{W} - \overset{\lower0.5em\hbox{$\smash{\scriptscriptstyle\frown}$}}{\tilde{T}}_{\infty } }},\overset{\lower0.5em\hbox{$\smash{\scriptscriptstyle\frown}$}}{\Theta } = \frac{{\overset{\lower0.5em\hbox{$\smash{\scriptscriptstyle\frown}$}}{\tilde{C}} - \overset{\lower0.5em\hbox{$\smash{\scriptscriptstyle\frown}$}}{\tilde{C}}_{\infty } }}{{\overset{\lower0.5em\hbox{$\smash{\scriptscriptstyle\frown}$}}{\tilde{C}}_{W} - \overset{\lower0.5em\hbox{$\smash{\scriptscriptstyle\frown}$}}{\tilde{C}}_{\infty } }},\overset{\lower0.5em\hbox{$\smash{\scriptscriptstyle\frown}$}}{\overset{\lower0.5em\hbox{$\smash{\scriptscriptstyle\smile}$}}{\lambda } }_{1} = \frac{{\overset{\lower0.5em\hbox{$\smash{\scriptscriptstyle\frown}$}}{\tilde{\lambda }}_{m} }}{{\tilde{t}_{0} }},\overset{\lower0.5em\hbox{$\smash{\scriptscriptstyle\frown}$}}{\overset{\lower0.5em\hbox{$\smash{\scriptscriptstyle\smile}$}}{\lambda } }_{2} = \frac{{\overset{\lower0.5em\hbox{$\smash{\scriptscriptstyle\frown}$}}{\tilde{\lambda }}_{o} }}{{\tilde{t}_{0} }}.} \right\}$$

Incorporating Eqs. (9), from Eqs. (1–7), we acquired the following linear PDEs

10 $$\begin{gathered} \ddot{\varphi }^{1} \left( {1 + \overset{\lower0.5em\hbox{$\smash{\scriptscriptstyle\frown}$}}{\overset{\lower0.5em\hbox{$\smash{\scriptscriptstyle\smile}$}}{\lambda } }_{1} \frac{\partial }{{\partial \overline{\tilde{t}}}}} \right)\frac{{\partial \overset{\lower0.5em\hbox{$\smash{\scriptscriptstyle\smile}$}}{U}^{ * } \left( {\overline{\tilde{Y}},\overline{\tilde{t}}} \right)}}{{\partial \overline{\tilde{t}}}} = \ddot{\varphi }^{0} \left( {1 + \overset{\lower0.5em\hbox{$\smash{\scriptscriptstyle\frown}$}}{\overset{\lower0.5em\hbox{$\smash{\scriptscriptstyle\smile}$}}{\lambda } }_{2} \frac{\partial }{{\partial \overline{\tilde{t}}}}} \right)\frac{{\partial \overset{\lower0.5em\hbox{$\smash{\scriptscriptstyle\smile}$}}{U}^{ * } \left( {\overline{\tilde{Y}},\overline{\tilde{t}}} \right)}}{{\partial \overline{\tilde{Y}}^{2} }} - \ddot{\varphi }^{2} \left( {1 + \overset{\lower0.5em\hbox{$\smash{\scriptscriptstyle\frown}$}}{\overset{\lower0.5em\hbox{$\smash{\scriptscriptstyle\smile}$}}{\lambda } }_{1} \frac{\partial }{{\partial \overline{\tilde{t}}}}} \right)\dddot M\overset{\lower0.5em\hbox{$\smash{\scriptscriptstyle\smile}$}}{U}^{ * } \left( {\overline{\tilde{Y}},\overline{\tilde{t}}} \right) \hfill \\ \, - \frac{1}{{\overset{\lower0.5em\hbox{$\smash{\scriptscriptstyle\smile}$}}{K} }}\ddot{\varphi }^{0} \left( {1 + \overset{\lower0.5em\hbox{$\smash{\scriptscriptstyle\frown}$}}{\overset{\lower0.5em\hbox{$\smash{\scriptscriptstyle\smile}$}}{\lambda } }_{2} \frac{\partial }{{\partial \overline{\tilde{t}}}}} \right)\overset{\lower0.5em\hbox{$\smash{\scriptscriptstyle\smile}$}}{U}^{ * } \left( {\overline{\tilde{Y}},\overline{\tilde{t}}} \right) + \ddot{\varphi }^{3} \left( {1 + \overset{\lower0.5em\hbox{$\smash{\scriptscriptstyle\frown}$}}{\overset{\lower0.5em\hbox{$\smash{\scriptscriptstyle\smile}$}}{\lambda } }_{1} \frac{\partial }{{\partial \overline{\tilde{t}}}}} \right)\hat{G}_{T} \overset{\lower0.5em\hbox{$\smash{\scriptscriptstyle\frown}$}}{\Phi } \left( {\overline{\tilde{Y}},\overline{\tilde{t}}} \right) \hfill \\ \, + \ddot{\varphi }^{4} \left( {1 + \overset{\lower0.5em\hbox{$\smash{\scriptscriptstyle\frown}$}}{\overset{\lower0.5em\hbox{$\smash{\scriptscriptstyle\smile}$}}{\lambda } }_{1} \frac{\partial }{{\partial \overline{\tilde{t}}}}} \right)\hat{G}_{C} \overset{\lower0.5em\hbox{$\smash{\scriptscriptstyle\frown}$}}{\Theta } \left( {\overline{\tilde{Y}},\overline{\tilde{t}}} \right), \hfill \\ \end{gathered}$$

11 $$\frac{{\ddot{\varphi }^{6} \Pr }}{{\ddot{\varphi }^{5} + R}}\frac{{\partial \overset{\lower0.5em\hbox{$\smash{\scriptscriptstyle\frown}$}}{\Phi } \left( {\overline{\tilde{Y}},\overline{\tilde{t}}} \right)}}{{\partial \overline{\tilde{t}}}} = \frac{{\partial^{2} \overset{\lower0.5em\hbox{$\smash{\scriptscriptstyle\frown}$}}{\Phi } \left( {\overline{\tilde{Y}},\overline{\tilde{t}}} \right)}}{{\partial \overline{\tilde{Y}}^{2} }},$$

12 $$Sc\frac{{\partial \overset{\lower0.5em\hbox{$\smash{\scriptscriptstyle\frown}$}}{\Theta } \left( {\overline{\tilde{Y}},\overline{\tilde{t}}} \right)}}{{\partial \overline{\tilde{t}}}} = \frac{{\partial^{2} \overset{\lower0.5em\hbox{$\smash{\scriptscriptstyle\frown}$}}{\Theta } \left( {\overline{\tilde{Y}},\overline{\tilde{t}}} \right)}}{{\partial \overline{\tilde{Y}}^{2} }},$$

and

13 $$\overset{\lower0.5em\hbox{$\smash{\scriptscriptstyle\smile}$}}{U}^{ * } \left( {\overline{\tilde{Y}},0} \right) = 0, \, \overset{\lower0.5em\hbox{$\smash{\scriptscriptstyle\frown}$}}{\Phi } \left( {\overline{\tilde{Y}},0} \right) = 0, \, \overset{\lower0.5em\hbox{$\smash{\scriptscriptstyle\frown}$}}{\Theta } \left( {\overline{\tilde{Y}},0} \right) = 0,\forall \overline{\tilde{Y}} \ge 0,$$

14 $$\left. \begin{gathered} \overset{\lower0.5em\hbox{$\smash{\scriptscriptstyle\smile}$}}{U}^{ * } \left( {0,\overline{\tilde{t}}} \right) = \left\{ \begin{gathered} \overline{\tilde{t}}; \, 0 \le \overline{\tilde{t}} < 1 \hfill \\ 1; \, 1 \le \overline{\tilde{t}} < \infty , \hfill \\ \end{gathered} \right. \hfill \\ \overset{\lower0.5em\hbox{$\smash{\scriptscriptstyle\frown}$}}{\Phi } \left( {0,\overline{\tilde{t}}} \right) = \left\{ \begin{gathered} \overline{\tilde{t}}; \, 0 \le \overline{\tilde{t}} < 1 \hfill \\ 1; \, 1 \le \overline{\tilde{t}} < \infty , \hfill \\ \end{gathered} \right. \hfill \\ \overset{\lower0.5em\hbox{$\smash{\scriptscriptstyle\frown}$}}{\Theta } \left( {0,\overline{\tilde{t}}} \right) = \left\{ \begin{gathered} \overline{\tilde{t}}; \, 0 \le \overline{\tilde{t}} < 1 \hfill \\ 1; \, 1 \le \overline{\tilde{t}} < \infty , \hfill \\ \end{gathered} \right. \hfill \\ \end{gathered} \right\}$$

15 $$\overset{\lower0.5em\hbox{$\smash{\scriptscriptstyle\smile}$}}{U}^{ * } \left( {\overline{\tilde{Y}},\overline{\tilde{t}}} \right) = 0, \, \overset{\lower0.5em\hbox{$\smash{\scriptscriptstyle\frown}$}}{\Phi } \left( {\overline{\tilde{Y}},\overline{\tilde{t}}} \right) = 0, \, \overset{\lower0.5em\hbox{$\smash{\scriptscriptstyle\frown}$}}{\Theta } \left( {\overline{\tilde{Y}},\overline{\tilde{t}}} \right) = 0,\forall \overline{\tilde{Y}} \to \infty .$$

where

16 $$\left. {\begin{array}{*{20}l} {\hat{G}_{T} = \frac{{\overset{\lower0.5em\hbox{$\smash{\scriptscriptstyle\frown}$}}{g} \left( {\overset{\lower0.5em\hbox{$\smash{\scriptscriptstyle\frown}$}}{\beta }_{T} \ddot{\upsilon }} \right)_{f} \left( {\overset{\lower0.5em\hbox{$\smash{\scriptscriptstyle\frown}$}}{\tilde{T}}_{W} - \overset{\lower0.5em\hbox{$\smash{\scriptscriptstyle\frown}$}}{\tilde{T}}_{\infty } } \right)}}{{\overset{\lower0.5em\hbox{$\smash{\scriptscriptstyle\frown}$}}{\tilde{u}}_{0}^{3} }},\overset{\lower0.5em\hbox{$\smash{\scriptscriptstyle\smile}$}}{K} = \frac{k}{{\tilde{t}_{0} \ddot{\upsilon }_{f} \overset{\lower0.5em\hbox{$\smash{\scriptscriptstyle\frown}$}}{\tilde{\phi }} }},\hat{G}_{C} = \frac{{\overset{\lower0.5em\hbox{$\smash{\scriptscriptstyle\frown}$}}{g} \left( {\overset{\lower0.5em\hbox{$\smash{\scriptscriptstyle\frown}$}}{\beta }_{C} \ddot{\upsilon }} \right)_{f} \left( {\overset{\lower0.5em\hbox{$\smash{\scriptscriptstyle\frown}$}}{\tilde{C}}_{W} - \overset{\lower0.5em\hbox{$\smash{\scriptscriptstyle\frown}$}}{\tilde{C}}_{\infty } } \right)}}{{\overset{\lower0.5em\hbox{$\smash{\scriptscriptstyle\frown}$}}{\tilde{u}}_{0}^{3} }},\dddot M = \frac{{\tilde{t}_{0} \overset{\lower0.5em\hbox{$\smash{\scriptscriptstyle\frown}$}}{\tilde{\sigma }}_{f} B_{0}^{2} }}{{\overset{\lower0.5em\hbox{$\smash{\scriptscriptstyle\frown}$}}{\tilde{\rho }}_{f} }},} \hfill \\ {\Pr = \left( {\frac{{\overset{\lower0.5em\hbox{$\smash{\scriptscriptstyle\frown}$}}{\tilde{\mu }} c_{p} }}{k}} \right)_{f} ,\ddot{\varphi }^{0} = \frac{1}{{\left( {1 - \ddot{\varphi }} \right)^{2.5} }},\ddot{\varphi }^{1} = \left( {1 - \ddot{\varphi }} \right) + \ddot{\varphi }\frac{{\overset{\lower0.5em\hbox{$\smash{\scriptscriptstyle\frown}$}}{\tilde{\rho }}_{s} }}{{\overset{\lower0.5em\hbox{$\smash{\scriptscriptstyle\frown}$}}{\tilde{\rho }}_{f} }},\ddot{\varphi }^{2} = \frac{{\overset{\lower0.5em\hbox{$\smash{\scriptscriptstyle\frown}$}}{\tilde{\sigma }}_{nf} }}{{\overset{\lower0.5em\hbox{$\smash{\scriptscriptstyle\frown}$}}{\tilde{\sigma }}_{f} }},\ddot{\varphi }^{3} = \left( {1 - \ddot{\varphi }} \right) + \ddot{\varphi }\frac{{\left( {\overset{\lower0.5em\hbox{$\smash{\scriptscriptstyle\frown}$}}{\beta }_{T} \overset{\lower0.5em\hbox{$\smash{\scriptscriptstyle\frown}$}}{\tilde{\rho }} } \right)_{s} }}{{\left( {\overset{\lower0.5em\hbox{$\smash{\scriptscriptstyle\frown}$}}{\beta }_{T} \overset{\lower0.5em\hbox{$\smash{\scriptscriptstyle\frown}$}}{\tilde{\rho }} } \right)_{f} }},} \hfill \\ {\tilde{R} = \frac{{16\tilde{\beta }^{ * } \overset{\lower0.5em\hbox{$\smash{\scriptscriptstyle\frown}$}}{\tilde{T}}_{\infty } }}{{3\overset{\lower0.5em\hbox{$\smash{\scriptscriptstyle\frown}$}}{\tilde{k}}_{f} \tilde{\delta }^{ * } }},Sc = \frac{{\dot{\upsilon }_{f} }}{{\overset{\lower0.5em\hbox{$\smash{\scriptscriptstyle\smile}$}}{D}_{f} }},\ddot{\varphi }^{4} = \left( {1 - \ddot{\varphi }} \right) + \ddot{\varphi }\frac{{\left( {\overset{\lower0.5em\hbox{$\smash{\scriptscriptstyle\frown}$}}{\beta }_{C} \overset{\lower0.5em\hbox{$\smash{\scriptscriptstyle\frown}$}}{\tilde{\rho }} } \right)_{s} }}{{\left( {\overset{\lower0.5em\hbox{$\smash{\scriptscriptstyle\frown}$}}{\beta }_{C} \overset{\lower0.5em\hbox{$\smash{\scriptscriptstyle\frown}$}}{\tilde{\rho }} } \right)_{f} }},\ddot{\varphi }^{5} = \frac{{\overset{\lower0.5em\hbox{$\smash{\scriptscriptstyle\frown}$}}{\tilde{k}}_{nf} }}{{\overset{\lower0.5em\hbox{$\smash{\scriptscriptstyle\frown}$}}{\tilde{k}}_{f} }},\ddot{\varphi }^{6} = \left( {1 - \ddot{\varphi }} \right) + \ddot{\varphi }\frac{{\left( {\overset{\lower0.5em\hbox{$\smash{\scriptscriptstyle\frown}$}}{\tilde{\rho }} c_{p} } \right)_{s} }}{{\left( {\overset{\lower0.5em\hbox{$\smash{\scriptscriptstyle\frown}$}}{\tilde{\rho }} c_{p} } \right)_{f} }}.} \hfill \\ \end{array} } \right\}$$

are the thermal Grashof number, the permeability of the permeable medium, mass Grashof number, magnetic factor, and, Prandtl number, respectively. Definition of Caputo fractional derivative and their LT are following

17 $$\left. \begin{gathered} ^{C}D_{{\overline{\tau }}}^{{\tilde{\gamma }}} \tilde{o}\left( {\overline{\tilde{Y}},\overline{\tau }} \right) = \frac{1}{{\Gamma \left( {\dot{v} - \tilde{\gamma }} \right)}}\int\limits_{0}^{{\overline{\tau }}} {\frac{{o^{{\left( {\dot{v}} \right)}} \left( {\overline{\tilde{Y}},\overline{\tau }} \right)}}{{\left( {\overline{\tau } - \tilde{s}} \right)}}} d\tilde{s}, \hfill \\ L\left\{ {{}^{C}D_{{\overline{\tau }}}^{{\tilde{\gamma }}} \tilde{o}\left( {\overline{\tilde{Y}},\overline{\tau }} \right)} \right\} = \overline{\tilde{o}}\left( {\overline{\tilde{Y}},\overset{\lower0.5em\hbox{$\smash{\scriptscriptstyle\smile}$}}{r} } \right) = \overset{\lower0.5em\hbox{$\smash{\scriptscriptstyle\smile}$}}{r}^{{\tilde{\gamma }}} L\left\{ {\tilde{o}\left( {\overline{\tilde{Y}},\overline{\tau }} \right)} \right\} - \overset{\lower0.5em\hbox{$\smash{\scriptscriptstyle\smile}$}}{r}^{{\tilde{\gamma } - 1}} \tilde{o}\left( {\overline{\tilde{Y}},0} \right). \hfill \\ \end{gathered} \right\}$$

The kernel of Caputo fractional derivative is singular if $$\overline{\tau } = \tilde{s}$$ and, $$\overset{\lower0.5em\hbox{$\smash{\scriptscriptstyle\smile}$}}{r}$$ is LT parameter. Without singular kernel, the definition of CF fractional derivative and their LT are following

18 $$\left. \begin{gathered} {}^{CF}D_{{\overline{\tau }}}^{{\tilde{\gamma }}} \tilde{o}\left( {\overline{\tilde{Y}},\overline{\tau }} \right) = \frac{1}{{\left( {\dot{v} - \tilde{\gamma }} \right)}}\int\limits_{0}^{{\overline{\tau }}} {e^{{\left( { - \frac{{\tilde{\gamma }\left( {\tilde{\gamma } - \overline{\tau }} \right)}}{{1 - \tilde{\gamma }}}} \right)}} \partial_{{\tilde{s}}} } \tilde{o}\left( {\overline{\tilde{Y}},\tilde{s}} \right)d\tilde{s}, \hfill \\ L\left\{ {{}^{CF}D_{{\overline{\tau }}}^{{\tilde{\gamma }}} \tilde{o}\left( {\overline{\tilde{Y}},\overline{\tau }} \right)} \right\} = \overline{\tilde{o}}\left( {\overline{\tilde{Y}},\overset{\lower0.5em\hbox{$\smash{\scriptscriptstyle\smile}$}}{r} } \right) = \frac{{\overset{\lower0.5em\hbox{$\smash{\scriptscriptstyle\smile}$}}{r} L\left\{ {\tilde{o}\left( {\overline{\tilde{Y}},\overline{\tau }} \right)} \right\} - \tilde{o}\left( {\overline{\tilde{Y}},0} \right)}}{{\left( {1 - \tilde{\gamma }} \right)\overset{\lower0.5em\hbox{$\smash{\scriptscriptstyle\smile}$}}{r} + \tilde{\gamma }}}. \hfill \\ \end{gathered} \right\}$$

Similarly, the definition of AB fractional derivative and their LT without singularity and locality are following

19 $$\left. \begin{gathered} {}^{AB}D_{{\overline{\tau }}}^{{\tilde{\gamma }}} \tilde{o}\left( {\overline{\tilde{Y}},\overline{\tau }} \right) = \frac{1}{{\left( {\dot{v} - \tilde{\gamma }} \right)}}\int\limits_{0}^{{\overline{\tau }}} {E_{{\tilde{\gamma }}}^{{\left( { - \frac{{\tilde{\gamma }\left( {\tilde{\gamma } - \overline{\tau }} \right)^{{\tilde{\gamma }}} }}{{1 - \tilde{\gamma }}}} \right)}} \partial_{{\tilde{s}}} } \tilde{o}\left( {\overline{\tilde{Y}},\tilde{s}} \right)d\tilde{s}, \hfill \\ L\left\{ {{}^{AB}D_{{\overline{\tau }}}^{{\tilde{\gamma }}} \tilde{o}\left( {\overline{\tilde{Y}},\overline{\tau }} \right)} \right\} = \overline{\tilde{o}}\left( {\overline{\tilde{Y}},\overset{\lower0.5em\hbox{$\smash{\scriptscriptstyle\smile}$}}{r} } \right) = \frac{{\overset{\lower0.5em\hbox{$\smash{\scriptscriptstyle\smile}$}}{r}^{{\tilde{\gamma }}} L\left\{ {\tilde{o}\left( {\overline{\tilde{Y}},\overline{\tau }} \right)} \right\} - \overset{\lower0.5em\hbox{$\smash{\scriptscriptstyle\smile}$}}{r}^{{\tilde{\gamma } - 1}} \tilde{o}\left( {\overline{\tilde{Y}},0} \right)}}{{\left( {1 - \tilde{\gamma }} \right)\overset{\lower0.5em\hbox{$\smash{\scriptscriptstyle\smile}$}}{r}^{{\tilde{\gamma }}} + \tilde{\gamma }}}. \hfill \\ \end{gathered} \right\}$$

Now, these three definitions of fractional derivative are being utilized to find the expressions of temperature, concentration and velocity profiles.

## Solutions of the temperature and concentration profile

**Figure 2:**
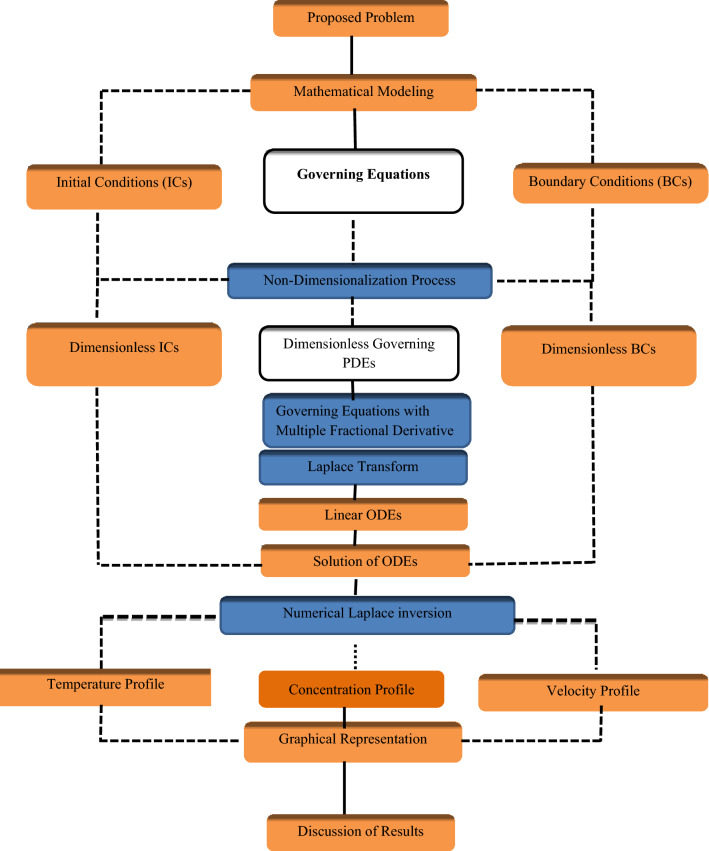
Methodology of the flow problem.

The solution of the problem is sought via the following methodology chart in Fig. 2

### Temperature profile using Caputo’s definition

Think time derivative in Eq. (11) as the Caputo’s fractional time derivative given by Eq. (17), then taking LT of resulting Eq. (11) and make use of respective transformed ICs and BCs, we get expression

20 $$\overline{\overset{\lower0.5em\hbox{$\smash{\scriptscriptstyle\frown}$}}{\Phi } }\left( {\overline{\tilde{Y}},\overset{\lower0.5em\hbox{$\smash{\scriptscriptstyle\smile}$}}{r} } \right) = \frac{{1 - e^{{ - \overset{\lower0.5em\hbox{$\smash{\scriptscriptstyle\smile}$}}{r} }} }}{{\overset{\lower0.5em\hbox{$\smash{\scriptscriptstyle\smile}$}}{r}^{2} }}e^{ - } \overline{\tilde{Y}}\sqrt {a_{0} \overset{\lower0.5em\hbox{$\smash{\scriptscriptstyle\smile}$}}{r}^{{\tilde{\alpha }}} } ,{\text{ where }}a_{0} = \frac{{\ddot{\varphi }^{6} \Pr }}{{\ddot{\varphi }^{5} + \tilde{R}}}.$$

### Temperature profile using Caputo-Fabrizio’s definition

Think time derivative in Eq. (11) as the Caputo-Fabrizio’s fractional time derivative given by Eq. (18), then taking LT of resulting Eq. (11) and make use of respective transformed ICs and BCs, we get expression

21 $$\overline{\overset{\lower0.5em\hbox{$\smash{\scriptscriptstyle\frown}$}}{\Phi } }\left( {\overline{\tilde{Y}},\overset{\lower0.5em\hbox{$\smash{\scriptscriptstyle\smile}$}}{r} } \right) = \frac{{1 - e^{{ - \overset{\lower0.5em\hbox{$\smash{\scriptscriptstyle\smile}$}}{r} }} }}{{\overset{\lower0.5em\hbox{$\smash{\scriptscriptstyle\smile}$}}{r}^{2} }}e^{{ - \overline{\tilde{Y}}}} \sqrt {\frac{{a_{0} \times b \times \overset{\lower0.5em\hbox{$\smash{\scriptscriptstyle\smile}$}}{r} }}{{\overset{\lower0.5em\hbox{$\smash{\scriptscriptstyle\smile}$}}{r} + b \times \tilde{\alpha }}}} ,\;\;\;{\text{where}}\;\;b = \frac{1}{{1 - \tilde{\alpha }}}.$$

### Temperature profile using Atangana-Baleanu’s definition

Think time derivative in Eq. (11) as the Atangana-Baleanu’s fractional time derivative given by Eq. (19), then taking LT of resulting Eq. (11) and make use of respective transformed ICs and BCs, we get expression

22 $$\overline{\overset{\lower0.5em\hbox{$\smash{\scriptscriptstyle\frown}$}}{\Phi } }\left( {\overline{\tilde{Y}},\overset{\lower0.5em\hbox{$\smash{\scriptscriptstyle\smile}$}}{r} } \right) = \frac{{1 - e^{{ - \overset{\lower0.5em\hbox{$\smash{\scriptscriptstyle\smile}$}}{r} }} }}{{\overset{\lower0.5em\hbox{$\smash{\scriptscriptstyle\smile}$}}{r}^{2} }}e^{{ - \overline{\tilde{Y}}}} \sqrt {\frac{{a_{0} \times b \times \overset{\lower0.5em\hbox{$\smash{\scriptscriptstyle\smile}$}}{r}^{{\tilde{\alpha }}} }}{{\overset{\lower0.5em\hbox{$\smash{\scriptscriptstyle\smile}$}}{r}^{{\tilde{\alpha }}} + b \times \tilde{\alpha }}}} .$$

In the absence of $\tilde{R}$, we get the solution of Saqib et al.^21^ and if $\tilde{\alpha } \to 1$ we acquire the solution of Seth et al.^55^.

### Concentration profile using Caputo’s definition

Think time derivative in Eq. (12) as the Caputo’s fractional time derivative given by Eq. (17), then taking LT of resulting Eq. (12) and make use of respective transformed ICs and BCs, we get expression

23 $$\overline{\overset{\lower0.5em\hbox{$\smash{\scriptscriptstyle\frown}$}}{\Theta } }\left( {\overline{\tilde{Y}},\overset{\lower0.5em\hbox{$\smash{\scriptscriptstyle\smile}$}}{r} } \right) = \frac{{1 - e^{{ - \overset{\lower0.5em\hbox{$\smash{\scriptscriptstyle\smile}$}}{r} }} }}{{\overset{\lower0.5em\hbox{$\smash{\scriptscriptstyle\smile}$}}{r}^{2} }}e^{{ - \overline{\tilde{Y}}}} \sqrt {Sc\overset{\lower0.5em\hbox{$\smash{\scriptscriptstyle\smile}$}}{r}^{{\tilde{\alpha }}} } .$$

### Concentration profile using Caputo-Fabrizio’s definition

Think time derivative in Eq. (12) as the Caputo-Fabrizio’s fractional time derivative given by Eq. (18), then taking LT of resulting Eq. (12) and make use of respective transformed ICs and BCs, we get expression

24 $$\overline{\tilde{\Theta }}\left( {\overline{\tilde{Y}},\overset{\lower0.5em\hbox{$\smash{\scriptscriptstyle\smile}$}}{r} } \right) = \frac{{1 - e^{{ - \overset{\lower0.5em\hbox{$\smash{\scriptscriptstyle\smile}$}}{r} }} }}{{\overset{\lower0.5em\hbox{$\smash{\scriptscriptstyle\smile}$}}{r}^{2} }}e^{{ - \overline{\tilde{Y}}}} \sqrt {\frac{{Sc \times b \times \overset{\lower0.5em\hbox{$\smash{\scriptscriptstyle\smile}$}}{r} }}{{\overset{\lower0.5em\hbox{$\smash{\scriptscriptstyle\smile}$}}{r} + b \times \tilde{\alpha }}}}$$

### Concentration profile using Atangana-Baleanu’s definition

Think time derivative in Eq. (12) as the Atangana-Baleanu’s fractional time derivative given by Eq. (19), then taking LT of resulting Eq. (12) and make use of respective transformed ICs and BCs, we get expression

25 $$\overline{\tilde{\Theta }}\left( {\overline{\tilde{Y}},\overset{\lower0.5em\hbox{$\smash{\scriptscriptstyle\smile}$}}{r} } \right) = \frac{{1 - e^{{ - \overset{\lower0.5em\hbox{$\smash{\scriptscriptstyle\smile}$}}{r} }} }}{{\overset{\lower0.5em\hbox{$\smash{\scriptscriptstyle\smile}$}}{r}^{2} }}e^{{ - \overline{\tilde{Y}}}} \sqrt {\frac{{Sc \times b \times \overset{\lower0.5em\hbox{$\smash{\scriptscriptstyle\smile}$}}{r}^{{\tilde{\alpha }}} }}{{\overset{\lower0.5em\hbox{$\smash{\scriptscriptstyle\smile}$}}{r}^{{\tilde{\alpha }}} + b \times \tilde{\alpha }}}}$$

## Velocity profile

Now, for velocity profile first we change the time derivative in Eq. (10) with fractional time derivative of orders $\tilde{\alpha }{\text{ and }}\tilde{\beta }$.

26 $$\begin{gathered} \ddot{\varphi }^{1} \left( {1 + \overset{\lower0.5em\hbox{$\smash{\scriptscriptstyle\frown}$}}{\overset{\lower0.5em\hbox{$\smash{\scriptscriptstyle\smile}$}}{\lambda } }_{1} D_{{\overline{\tilde{t}}}}^{{\tilde{\alpha }}} } \right)D_{{\overline{\tilde{t}}}}^{{\tilde{\alpha }}} \overset{\lower0.5em\hbox{$\smash{\scriptscriptstyle\smile}$}}{U}^{ * } \left( {\overline{\tilde{Y}},\overline{\tilde{t}}} \right) = \ddot{\varphi }^{0} \left( {1 + \overset{\lower0.5em\hbox{$\smash{\scriptscriptstyle\frown}$}}{\overset{\lower0.5em\hbox{$\smash{\scriptscriptstyle\smile}$}}{\lambda } }_{2} D_{{\overline{\tilde{t}}}}^{{\tilde{\beta }}} } \right)\partial_{{\overline{\tilde{Y}}\overline{\tilde{Y}}}}^{2} \overset{\lower0.5em\hbox{$\smash{\scriptscriptstyle\smile}$}}{U}^{ * } \left( {\overline{\tilde{Y}},\overline{\tilde{t}}} \right) - \ddot{\varphi }^{2} \left( {1 + \overset{\lower0.5em\hbox{$\smash{\scriptscriptstyle\frown}$}}{\overset{\lower0.5em\hbox{$\smash{\scriptscriptstyle\smile}$}}{\lambda } }_{1} D_{{\overline{\tilde{t}}}}^{{\tilde{\alpha }}} } \right)\dddot M\overset{\lower0.5em\hbox{$\smash{\scriptscriptstyle\smile}$}}{U}^{ * } \left( {\overline{\tilde{Y}},\overline{\tilde{t}}} \right) \hfill \\ \, - \frac{1}{{\overset{\lower0.5em\hbox{$\smash{\scriptscriptstyle\smile}$}}{K} }}\ddot{\varphi }^{0} \left( {1 + \overset{\lower0.5em\hbox{$\smash{\scriptscriptstyle\frown}$}}{\overset{\lower0.5em\hbox{$\smash{\scriptscriptstyle\smile}$}}{\lambda } }_{2} D_{{\overline{\tilde{t}}}}^{{\tilde{\beta }}} } \right)\overset{\lower0.5em\hbox{$\smash{\scriptscriptstyle\smile}$}}{U}^{ * } \left( {\overline{\tilde{Y}},\overline{\tilde{t}}} \right) + \ddot{\varphi }^{3} \left( {1 + \overset{\lower0.5em\hbox{$\smash{\scriptscriptstyle\frown}$}}{\overset{\lower0.5em\hbox{$\smash{\scriptscriptstyle\smile}$}}{\lambda } }_{1} D_{{\overline{\tilde{t}}}}^{{\tilde{\alpha }}} } \right)\hat{G}_{T} \overset{\lower0.5em\hbox{$\smash{\scriptscriptstyle\frown}$}}{\Phi } \left( {\overline{\tilde{Y}},\overline{\tilde{t}}} \right) \hfill \\ \, + \ddot{\varphi }^{4} \left( {1 + \overset{\lower0.5em\hbox{$\smash{\scriptscriptstyle\frown}$}}{\overset{\lower0.5em\hbox{$\smash{\scriptscriptstyle\smile}$}}{\lambda } }_{1} D_{{\overline{\tilde{t}}}}^{{\tilde{\alpha }}} } \right)\hat{G}_{C} \overset{\lower0.5em\hbox{$\smash{\scriptscriptstyle\frown}$}}{\Theta } \left( {\overline{\tilde{Y}},\overline{\tilde{t}}} \right). \hfill \\ \end{gathered}$$

### Velocity profile via Caputo approach

Think time derivative in Eq. (26) as the Caputo’s fractional time derivative given by Eq. (17), then taking LT of resulting Eq. (26) and make use of respective transformed ICs and BCs along with Eqs. (20) and (23), we get the expression

27 $$\begin{aligned} \overset{\lower0.5em\hbox{$\smash{\scriptscriptstyle\smile}$}}{U}^{ * } \left( {\overline{\tilde{Y}},\overset{\lower0.5em\hbox{$\smash{\scriptscriptstyle\smile}$}}{r} } \right) = & \left[ {\frac{{1 - e^{{ - \overset{\lower0.5em\hbox{$\smash{\scriptscriptstyle\smile}$}}{r} }} }}{{\overset{\lower0.5em\hbox{$\smash{\scriptscriptstyle\smile}$}}{r}^{2} }}} \right] \times e^{{ - \overline{\tilde{Y}}\sqrt {\overline{\Omega }\left( {\overset{\lower0.5em\hbox{$\smash{\scriptscriptstyle\smile}$}}{r} } \right)\left( {\dddot M_{0} + b_{0} \overset{\lower0.5em\hbox{$\smash{\scriptscriptstyle\smile}$}}{r}^{{\tilde{\alpha }}} } \right) + \frac{1}{{\overset{\lower0.5em\hbox{$\smash{\scriptscriptstyle\smile}$}}{K} }}} }} \\ & \;\; + \left[ {\frac{{\overset{\lower0.5em\hbox{$\smash{\scriptscriptstyle\frown}$}}{G}_{T}^{0} \times \overline{\Omega }\left( {\overset{\lower0.5em\hbox{$\smash{\scriptscriptstyle\smile}$}}{r} } \right)\left( {1 - e^{{ - \overset{\lower0.5em\hbox{$\smash{\scriptscriptstyle\smile}$}}{r} }} } \right)}}{{\overset{\lower0.5em\hbox{$\smash{\scriptscriptstyle\smile}$}}{r}^{2} \left[ {a_{0} \overset{\lower0.5em\hbox{$\smash{\scriptscriptstyle\smile}$}}{r}^{{\tilde{\alpha }}} - \left( {\overline{\Omega }\left( {\overset{\lower0.5em\hbox{$\smash{\scriptscriptstyle\smile}$}}{r} } \right)\left( {\dddot M_{0} + b_{0} \overset{\lower0.5em\hbox{$\smash{\scriptscriptstyle\smile}$}}{r}^{{\tilde{\alpha }}} } \right) + \frac{1}{{\overset{\lower0.5em\hbox{$\smash{\scriptscriptstyle\smile}$}}{K} }}} \right)} \right]}}} \right] \times \left[ {e^{{ - \overline{\tilde{Y}}\sqrt {\overline{\Omega }\left( {\overset{\lower0.5em\hbox{$\smash{\scriptscriptstyle\smile}$}}{r} } \right)\left( {\dddot M_{0} + b_{0} \overset{\lower0.5em\hbox{$\smash{\scriptscriptstyle\smile}$}}{r}^{{\tilde{\alpha }}} } \right) + \frac{1}{{\overset{\lower0.5em\hbox{$\smash{\scriptscriptstyle\smile}$}}{K} }}} }} - e^{{ - \overline{\tilde{Y}}\sqrt {a_{0} \overset{\lower0.5em\hbox{$\smash{\scriptscriptstyle\smile}$}}{r}^{{\tilde{\alpha }}} } }} } \right] \\ & \;\;{ + }\left[ {\frac{{\overset{\lower0.5em\hbox{$\smash{\scriptscriptstyle\frown}$}}{G}_{C}^{1} \times \overline{\Omega }\left( {\overset{\lower0.5em\hbox{$\smash{\scriptscriptstyle\smile}$}}{r} } \right)\left( {1 - e^{{ - \overset{\lower0.5em\hbox{$\smash{\scriptscriptstyle\smile}$}}{r} }} } \right)}}{{\overset{\lower0.5em\hbox{$\smash{\scriptscriptstyle\smile}$}}{r}^{2} \left[ {Sc\overset{\lower0.5em\hbox{$\smash{\scriptscriptstyle\smile}$}}{r}^{{\tilde{\alpha }}} - \left( {\overline{\Omega }\left( {\overset{\lower0.5em\hbox{$\smash{\scriptscriptstyle\smile}$}}{r} } \right)\left( {\dddot M_{0} + b_{0} \overset{\lower0.5em\hbox{$\smash{\scriptscriptstyle\smile}$}}{r}^{{\tilde{\alpha }}} } \right) + \frac{1}{{\overset{\lower0.5em\hbox{$\smash{\scriptscriptstyle\smile}$}}{K} }}} \right)} \right]}}} \right] \times \left[ {e^{{ - \overline{\tilde{Y}}\sqrt {\overline{\Omega }\left( {\overset{\lower0.5em\hbox{$\smash{\scriptscriptstyle\smile}$}}{r} } \right)\left( {\dddot M_{0} + b_{0} \overset{\lower0.5em\hbox{$\smash{\scriptscriptstyle\smile}$}}{r}^{{\tilde{\alpha }}} } \right) + \frac{1}{{\overset{\lower0.5em\hbox{$\smash{\scriptscriptstyle\smile}$}}{K} }}} }} - e^{{ - \overline{\tilde{Y}}\sqrt {Sc\overset{\lower0.5em\hbox{$\smash{\scriptscriptstyle\smile}$}}{r}^{{\tilde{\alpha }}} } }} } \right], \\ \end{aligned}$$

where $$\overline{\Omega }\left( {\overset{\lower0.5em\hbox{$\smash{\scriptscriptstyle\smile}$}}{r} } \right) = \frac{{\left( {1 + \overset{\lower0.5em\hbox{$\smash{\scriptscriptstyle\frown}$}}{\overset{\lower0.5em\hbox{$\smash{\scriptscriptstyle\smile}$}}{\lambda } }_{1} \overset{\lower0.5em\hbox{$\smash{\scriptscriptstyle\smile}$}}{r}^{{\tilde{\alpha }}} } \right)}}{{\left( {1 + \overset{\lower0.5em\hbox{$\smash{\scriptscriptstyle\frown}$}}{\overset{\lower0.5em\hbox{$\smash{\scriptscriptstyle\smile}$}}{\lambda } }_{2} \overset{\lower0.5em\hbox{$\smash{\scriptscriptstyle\smile}$}}{r}^{{\tilde{\beta }}} } \right)}},\hat{G}_{T}^{0} = \frac{{\ddot{\varphi }^{3} }}{{\ddot{\varphi }^{0} }}\hat{G}_{T} ,\hat{G}_{C}^{1} = \frac{{\ddot{\varphi }^{4} }}{{\ddot{\varphi }^{0} }}\hat{G}_{T} ,\dddot M_{0} = \frac{{\ddot{\varphi }^{2} }}{{\ddot{\varphi }^{0} }}M,b_{0} = \frac{{\ddot{\varphi }^{1} }}{{\ddot{\varphi }^{0} }}$$.

### Velocity profile using Caputo-Fabrizio’s definition

Think time derivative in Eq. (26) as the Caputo-Fabrizio’s fractional time derivative given by Eq. (18), then taking LT of resulting Eq. (26) and make use of respective transformed ICs and BCs along with Eqs. (21) and (24), we get the expression

28 $$\begin{aligned} \overset{\lower0.5em\hbox{$\smash{\scriptscriptstyle\smile}$}}{U}^{ * } \left( {\overline{\tilde{Y}},\overset{\lower0.5em\hbox{$\smash{\scriptscriptstyle\smile}$}}{r} } \right) = & \left[ {\frac{{1 - e^{{ - \overset{\lower0.5em\hbox{$\smash{\scriptscriptstyle\smile}$}}{r} }} }}{{\overset{\lower0.5em\hbox{$\smash{\scriptscriptstyle\smile}$}}{r}^{2} }}} \right] \times e^{{ - \overline{\tilde{Y}}\sqrt {\frac{{\hat{B}\left( {\overset{\lower0.5em\hbox{$\smash{\scriptscriptstyle\smile}$}}{r} } \right)\left( {\dddot M_{0} + b_{0} \overset{\lower0.5em\hbox{$\smash{\scriptscriptstyle\smile}$}}{r} } \right)}}{{\hat{A}\left( {\overset{\lower0.5em\hbox{$\smash{\scriptscriptstyle\smile}$}}{r} } \right)}} + \frac{1}{{\overset{\lower0.5em\hbox{$\smash{\scriptscriptstyle\smile}$}}{K} }}} }} \\ & \;\; + \left[ {\frac{{\hat{G}_{T}^{0} \hat{B}\left( {\overset{\lower0.5em\hbox{$\smash{\scriptscriptstyle\smile}$}}{r} } \right)\left( {1 - e^{{ - \overset{\lower0.5em\hbox{$\smash{\scriptscriptstyle\smile}$}}{r} }} } \right)}}{{\overset{\lower0.5em\hbox{$\smash{\scriptscriptstyle\smile}$}}{r}^{2} \hat{A}\left( {\overset{\lower0.5em\hbox{$\smash{\scriptscriptstyle\smile}$}}{r} } \right)\left[ {\frac{{a_{0} \times b \times \overset{\lower0.5em\hbox{$\smash{\scriptscriptstyle\smile}$}}{r} }}{{\overset{\lower0.5em\hbox{$\smash{\scriptscriptstyle\smile}$}}{r} + b \times \tilde{\alpha }}} - \left[ {\frac{{\hat{B}\left( {\overset{\lower0.5em\hbox{$\smash{\scriptscriptstyle\smile}$}}{r} } \right)\left( {\dddot M_{0} + b_{0} \overset{\lower0.5em\hbox{$\smash{\scriptscriptstyle\smile}$}}{r} } \right)}}{{\hat{A}\left( {\overset{\lower0.5em\hbox{$\smash{\scriptscriptstyle\smile}$}}{r} } \right)}} + \frac{1}{{\overset{\lower0.5em\hbox{$\smash{\scriptscriptstyle\smile}$}}{K} }}} \right]} \right]}}} \right] \times \left[ {e^{{ - \overline{\tilde{Y}}\sqrt {\frac{{\hat{B}\left( {\overset{\lower0.5em\hbox{$\smash{\scriptscriptstyle\smile}$}}{r} } \right)\left( {\dddot M_{0} + b_{0} \overset{\lower0.5em\hbox{$\smash{\scriptscriptstyle\smile}$}}{r} } \right)}}{{\hat{A}\left( {\overset{\lower0.5em\hbox{$\smash{\scriptscriptstyle\smile}$}}{r} } \right)}} + \frac{1}{{\overset{\lower0.5em\hbox{$\smash{\scriptscriptstyle\smile}$}}{K} }}} }} - e^{{ - \overline{\tilde{Y}}\sqrt {\frac{{a_{0} \times b \times \overset{\lower0.5em\hbox{$\smash{\scriptscriptstyle\smile}$}}{r} }}{{\overset{\lower0.5em\hbox{$\smash{\scriptscriptstyle\smile}$}}{r} + b \times \tilde{\alpha }}}} }} } \right] \\ & \;\; + \left[ {\frac{{\hat{G}_{C}^{1} \hat{B}\left( {\overset{\lower0.5em\hbox{$\smash{\scriptscriptstyle\smile}$}}{r} } \right)\left( {1 - e^{{ - \overset{\lower0.5em\hbox{$\smash{\scriptscriptstyle\smile}$}}{r} }} } \right)}}{{\overset{\lower0.5em\hbox{$\smash{\scriptscriptstyle\smile}$}}{r}^{2} \hat{A}\left( {\overset{\lower0.5em\hbox{$\smash{\scriptscriptstyle\smile}$}}{r} } \right)\left[ {\frac{{Sc \times b \times \overset{\lower0.5em\hbox{$\smash{\scriptscriptstyle\smile}$}}{r} }}{{\overset{\lower0.5em\hbox{$\smash{\scriptscriptstyle\smile}$}}{r} + b \times \tilde{\alpha }}} - \left[ {\frac{{\hat{B}\left( {\overset{\lower0.5em\hbox{$\smash{\scriptscriptstyle\smile}$}}{r} } \right)\left( {\dddot M_{0} + b_{0} \overset{\lower0.5em\hbox{$\smash{\scriptscriptstyle\smile}$}}{r} } \right)}}{{\hat{A}\left( {\overset{\lower0.5em\hbox{$\smash{\scriptscriptstyle\smile}$}}{r} } \right)}} + \frac{1}{{\overset{\lower0.5em\hbox{$\smash{\scriptscriptstyle\smile}$}}{K} }}} \right]} \right]}}} \right] \times \left[ {e^{{ - \overline{\tilde{Y}}\sqrt {\frac{{\hat{B}\left( {\overset{\lower0.5em\hbox{$\smash{\scriptscriptstyle\smile}$}}{r} } \right)\left( {\dddot M_{0} + b_{0} \overset{\lower0.5em\hbox{$\smash{\scriptscriptstyle\smile}$}}{r} } \right)}}{{\hat{A}\left( {\overset{\lower0.5em\hbox{$\smash{\scriptscriptstyle\smile}$}}{r} } \right)}} + \frac{1}{{\overset{\lower0.5em\hbox{$\smash{\scriptscriptstyle\smile}$}}{K} }}} }} - e^{{ - \overline{\tilde{Y}}\sqrt {\frac{{Sc \times b \times \overset{\lower0.5em\hbox{$\smash{\scriptscriptstyle\smile}$}}{r} }}{{\overset{\lower0.5em\hbox{$\smash{\scriptscriptstyle\smile}$}}{r} + b \times \tilde{\alpha }}}} }} } \right], \\ \end{aligned}$$

where;$$\hat{A}\left( {\overset{\lower0.5em\hbox{$\smash{\scriptscriptstyle\smile}$}}{r} } \right) = \frac{{\left( {1 - \tilde{\beta }} \right)\overset{\lower0.5em\hbox{$\smash{\scriptscriptstyle\smile}$}}{r} + \tilde{\beta } + \overset{\lower0.5em\hbox{$\smash{\scriptscriptstyle\frown}$}}{\overset{\lower0.5em\hbox{$\smash{\scriptscriptstyle\smile}$}}{\lambda } }_{2} \overset{\lower0.5em\hbox{$\smash{\scriptscriptstyle\smile}$}}{r} }}{{\left( {1 - \tilde{\beta }} \right)\overset{\lower0.5em\hbox{$\smash{\scriptscriptstyle\smile}$}}{r} + \tilde{\beta }}},\hat{B}\left( {\overset{\lower0.5em\hbox{$\smash{\scriptscriptstyle\smile}$}}{r} } \right) = \frac{{\left( {1 - \tilde{\alpha }} \right)\overset{\lower0.5em\hbox{$\smash{\scriptscriptstyle\smile}$}}{r} + \tilde{\alpha } + \overset{\lower0.5em\hbox{$\smash{\scriptscriptstyle\frown}$}}{\overset{\lower0.5em\hbox{$\smash{\scriptscriptstyle\smile}$}}{\lambda } }_{1} \overset{\lower0.5em\hbox{$\smash{\scriptscriptstyle\smile}$}}{r} }}{{\left( {1 - \tilde{\alpha }} \right)\overset{\lower0.5em\hbox{$\smash{\scriptscriptstyle\smile}$}}{r} + \tilde{\alpha }}}.$$

### Velocity profile using Atangana-Baleanu’s definition

Think time derivative in Eq. (26) as the Atangana-Baleanu’s fractional time derivative given by Eq. (19), then taking LT of resulting Eq. (26) and make use of respective transformed ICs and BCs along with Eqs. (22) and (25), we get the expression

29 $$\begin{aligned} \overset{\lower0.5em\hbox{$\smash{\scriptscriptstyle\smile}$}}{U}^{ * } \left( {\overline{\tilde{Y}},\overset{\lower0.5em\hbox{$\smash{\scriptscriptstyle\smile}$}}{r} } \right) = & \left[ {\frac{{1 - e^{{ - \overset{\lower0.5em\hbox{$\smash{\scriptscriptstyle\smile}$}}{r} }} }}{{\overset{\lower0.5em\hbox{$\smash{\scriptscriptstyle\smile}$}}{r}^{2} }}} \right] \times e^{{ - \overline{\tilde{Y}}\sqrt {\frac{{\hat{B}_{11} \left( {\overset{\lower0.5em\hbox{$\smash{\scriptscriptstyle\smile}$}}{r} } \right)\left( {\dddot M_{0} + b_{0} \overset{\lower0.5em\hbox{$\smash{\scriptscriptstyle\smile}$}}{r} } \right)}}{{\hat{A}_{11} \left( {\overset{\lower0.5em\hbox{$\smash{\scriptscriptstyle\smile}$}}{r} } \right)}} + \frac{1}{{\overset{\lower0.5em\hbox{$\smash{\scriptscriptstyle\smile}$}}{K} }}} }} \\ & \;\; + \left[ {\frac{{\hat{G}_{T}^{0} \hat{B}_{11} \left( {\overset{\lower0.5em\hbox{$\smash{\scriptscriptstyle\smile}$}}{r} } \right)\left( {1 - e^{{ - \overset{\lower0.5em\hbox{$\smash{\scriptscriptstyle\smile}$}}{r} }} } \right)}}{{\overset{\lower0.5em\hbox{$\smash{\scriptscriptstyle\smile}$}}{r}^{2} \hat{A}_{11} \left( {\overset{\lower0.5em\hbox{$\smash{\scriptscriptstyle\smile}$}}{r} } \right)\left[ {\frac{{a_{0} \times b \times \overset{\lower0.5em\hbox{$\smash{\scriptscriptstyle\smile}$}}{r}^{{\tilde{\alpha }}} }}{{\overset{\lower0.5em\hbox{$\smash{\scriptscriptstyle\smile}$}}{r}^{{\tilde{\alpha }}} + b \times \tilde{\alpha }}} - \left[ {\frac{{\hat{B}_{11} \left( {\overset{\lower0.5em\hbox{$\smash{\scriptscriptstyle\smile}$}}{r} } \right)\left( {\dddot M_{0} + b_{0} \overset{\lower0.5em\hbox{$\smash{\scriptscriptstyle\smile}$}}{r} } \right)}}{{\hat{A}_{11} \left( {\overset{\lower0.5em\hbox{$\smash{\scriptscriptstyle\smile}$}}{r} } \right)}} + \frac{1}{{\overset{\lower0.5em\hbox{$\smash{\scriptscriptstyle\smile}$}}{K} }}} \right]} \right]}}} \right] \times \left[ {e^{{ - \overline{\tilde{Y}}\sqrt {\frac{{\hat{B}_{11} \left( {\overset{\lower0.5em\hbox{$\smash{\scriptscriptstyle\smile}$}}{r} } \right)\left( {\dddot M_{0} + b_{0} \overset{\lower0.5em\hbox{$\smash{\scriptscriptstyle\smile}$}}{r} } \right)}}{{\hat{A}_{11} \left( {\overset{\lower0.5em\hbox{$\smash{\scriptscriptstyle\smile}$}}{r} } \right)}} + \frac{1}{{\overset{\lower0.5em\hbox{$\smash{\scriptscriptstyle\smile}$}}{K} }}} }} - e^{{ - \overline{\tilde{Y}}\sqrt {\frac{{a_{0} \times b \times \overset{\lower0.5em\hbox{$\smash{\scriptscriptstyle\smile}$}}{r}^{{\tilde{\alpha }}} }}{{\overset{\lower0.5em\hbox{$\smash{\scriptscriptstyle\smile}$}}{r}^{{\tilde{\alpha }}} + b \times \tilde{\alpha }}}} }} } \right] \\ & \;\; + \left[ {\frac{{\hat{G}_{C}^{1} \hat{B}_{11} \left( {\overset{\lower0.5em\hbox{$\smash{\scriptscriptstyle\smile}$}}{r} } \right)\left( {1 - e^{{ - \overset{\lower0.5em\hbox{$\smash{\scriptscriptstyle\smile}$}}{r} }} } \right)}}{{\overset{\lower0.5em\hbox{$\smash{\scriptscriptstyle\smile}$}}{r}^{2} \hat{A}_{11} \left( {\overset{\lower0.5em\hbox{$\smash{\scriptscriptstyle\smile}$}}{r} } \right)\left[ {\frac{{Sc \times b \times \overset{\lower0.5em\hbox{$\smash{\scriptscriptstyle\smile}$}}{r}^{{\tilde{\alpha }}} }}{{\overset{\lower0.5em\hbox{$\smash{\scriptscriptstyle\smile}$}}{r}^{{\tilde{\alpha }}} + b \times \tilde{\alpha }}} - \left[ {\frac{{\hat{B}_{11} \left( {\overset{\lower0.5em\hbox{$\smash{\scriptscriptstyle\smile}$}}{r} } \right)\left( {\dddot M_{0} + b_{0} \overset{\lower0.5em\hbox{$\smash{\scriptscriptstyle\smile}$}}{r} } \right)}}{{\hat{A}_{11} \left( {\overset{\lower0.5em\hbox{$\smash{\scriptscriptstyle\smile}$}}{r} } \right)}} + \frac{1}{{\overset{\lower0.5em\hbox{$\smash{\scriptscriptstyle\smile}$}}{K} }}} \right]} \right]}}} \right] \times \left[ {e^{{ - \overline{\tilde{Y}}\sqrt {\frac{{\hat{B}_{11} \left( {\overset{\lower0.5em\hbox{$\smash{\scriptscriptstyle\smile}$}}{r} } \right)\left( {\dddot M_{0} + b_{0} \overset{\lower0.5em\hbox{$\smash{\scriptscriptstyle\smile}$}}{r} } \right)}}{{\hat{A}_{11} \left( {\overset{\lower0.5em\hbox{$\smash{\scriptscriptstyle\smile}$}}{r} } \right)}} + \frac{1}{{\overset{\lower0.5em\hbox{$\smash{\scriptscriptstyle\smile}$}}{K} }}} }} - e^{{ - \overline{\tilde{Y}}\sqrt {\frac{{Sc \times b \times \overset{\lower0.5em\hbox{$\smash{\scriptscriptstyle\smile}$}}{r}^{{\tilde{\alpha }}} }}{{\overset{\lower0.5em\hbox{$\smash{\scriptscriptstyle\smile}$}}{r}^{{\tilde{\alpha }}} + b \times \tilde{\alpha }}}} }} } \right], \\ \end{aligned}$$

where $$\hat{A}_{11} \left( {\overset{\lower0.5em\hbox{$\smash{\scriptscriptstyle\smile}$}}{r} } \right) = \frac{{\left( {1 - \tilde{\beta }} \right)\overset{\lower0.5em\hbox{$\smash{\scriptscriptstyle\smile}$}}{r}^{{\tilde{\beta }}} + \tilde{\beta } + \overset{\lower0.5em\hbox{$\smash{\scriptscriptstyle\frown}$}}{\overset{\lower0.5em\hbox{$\smash{\scriptscriptstyle\smile}$}}{\lambda } }_{2} \overset{\lower0.5em\hbox{$\smash{\scriptscriptstyle\smile}$}}{r}^{{\tilde{\beta }}} }}{{\left( {1 - \tilde{\beta }} \right)\overset{\lower0.5em\hbox{$\smash{\scriptscriptstyle\smile}$}}{r}^{{\tilde{\beta }}} + \tilde{\beta }}},\,\,\hat{B}_{11} \left( {\overset{\lower0.5em\hbox{$\smash{\scriptscriptstyle\smile}$}}{r} } \right) = \frac{{\left( {1 - \tilde{\alpha }} \right)\overset{\lower0.5em\hbox{$\smash{\scriptscriptstyle\smile}$}}{r}^{{\tilde{\alpha }}} + \tilde{\alpha } + \overset{\lower0.5em\hbox{$\smash{\scriptscriptstyle\frown}$}}{\overset{\lower0.5em\hbox{$\smash{\scriptscriptstyle\smile}$}}{\lambda } }_{1} \overset{\lower0.5em\hbox{$\smash{\scriptscriptstyle\smile}$}}{r}^{{\tilde{\alpha }}} }}{{\left( {1 - \tilde{\alpha }} \right)\overset{\lower0.5em\hbox{$\smash{\scriptscriptstyle\smile}$}}{r}^{{\tilde{\alpha }}} + \tilde{\alpha }}}.$$

If $$\tilde{\alpha } \to 1{\text{ and }}\tilde{\beta } \to 1$$ then non-integer model is reduced to integer-order (classical model). Further, if $$\tilde{R} \to 0{\text{ and }}\hat{G}_{C}^{1} \to 0$$, then the flow problem reduces to the problem of Saqib et al.^9^. We discuss some results as a special case, like if $$\overset{\lower0.5em\hbox{$\smash{\scriptscriptstyle\frown}$}}{\overset{\lower0.5em\hbox{$\smash{\scriptscriptstyle\smile}$}}{\lambda } }_{2} \to 0$$, then we obtain the solution for Maxwell nanofluid and also if $$\overset{\lower0.5em\hbox{$\smash{\scriptscriptstyle\frown}$}}{\overset{\lower0.5em\hbox{$\smash{\scriptscriptstyle\smile}$}}{\lambda } }_{1} \to 0$$ and $$\overset{\lower0.5em\hbox{$\smash{\scriptscriptstyle\frown}$}}{\overset{\lower0.5em\hbox{$\smash{\scriptscriptstyle\smile}$}}{\lambda } }_{2} \ne 0$$, the solution for second-grade nanofluid is acquired. For Newtonian fluid both the times, relaxation and retardation, must be zero. Due to the complex combination of Laplace transform in Eqs. (20–29), it is not very easy to compute the inverse Laplace analytically, so now we utilize numerical algorithms for the inversion of Laplace numerically, like of Zakian’s and Stehfest’s. Also, we will present the comparison of these two algorithms in tabular form.

## Results and discussion

In this article we investigated the heat and mass transfer of MHD Oldroyd-B fluid with ramped conditions. The Oldroyd-B fluid is taken as a base fluid (Blood) with a suspension of gold nano-particles, to make the solution of non-Newtonian bio-magnetic nanofluid. The surface medium is taken as porous. The well-known equation of Oldroyd-B nano-fluid of integer order derivative has been generalized to a non-integer order derivative. Three different types of definitions of fractional differential operators, like Caputo, Caputo-Fabrizio, Atangana-Baleanu are used to develop the resulting fractional nano-fluid model. The graphs for related parameters are plotted via MATLAB. Laplace transform technique is utilized for the solution of temperature, concentration, and velocity distributions, and for inversion purposes numerical algorithms for the inversion of Laplace numerically, like of Zakian’s and Stehfest’s are utilized. Their comparison is also presented in tabular form. The range of plots for various parameters is taken from 0 to 10.

**Figure 3:**
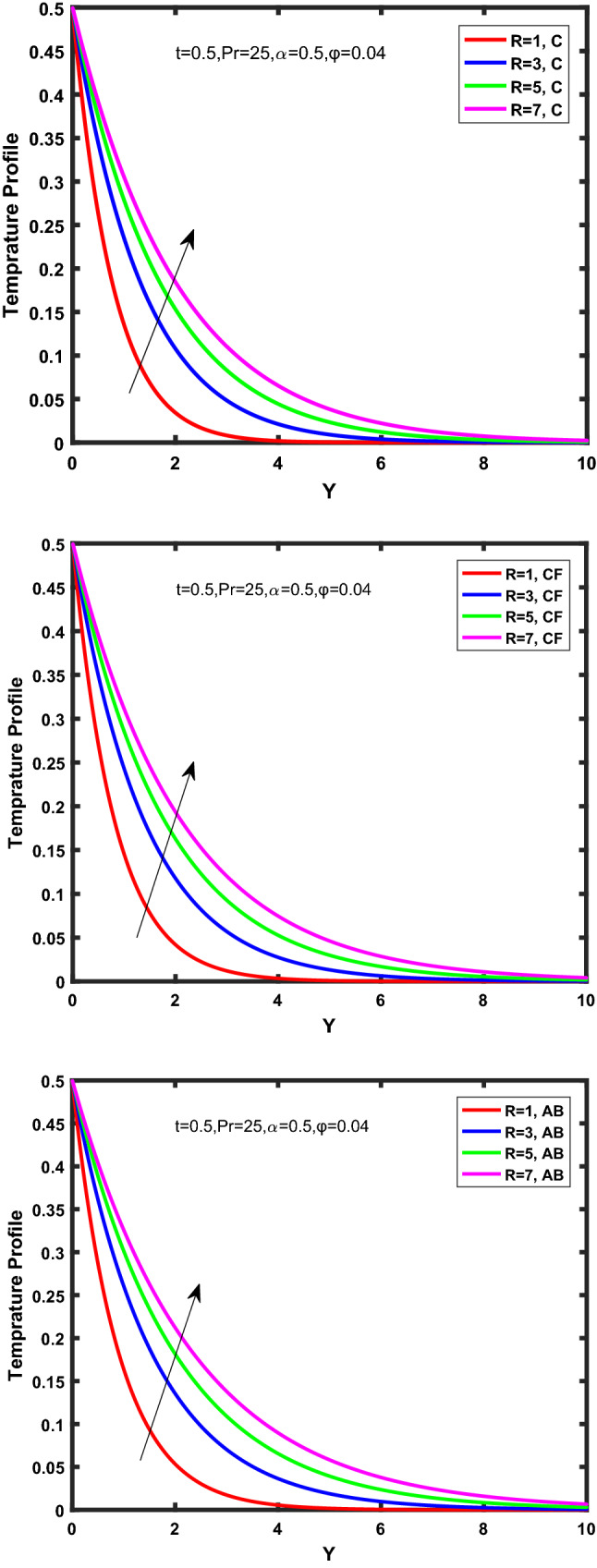
Temperature profile of Caputo, Caputo-Fabrizio, and Atangana-Baleanu for radiation factor $\tilde{R}$.

**Figure 4:**
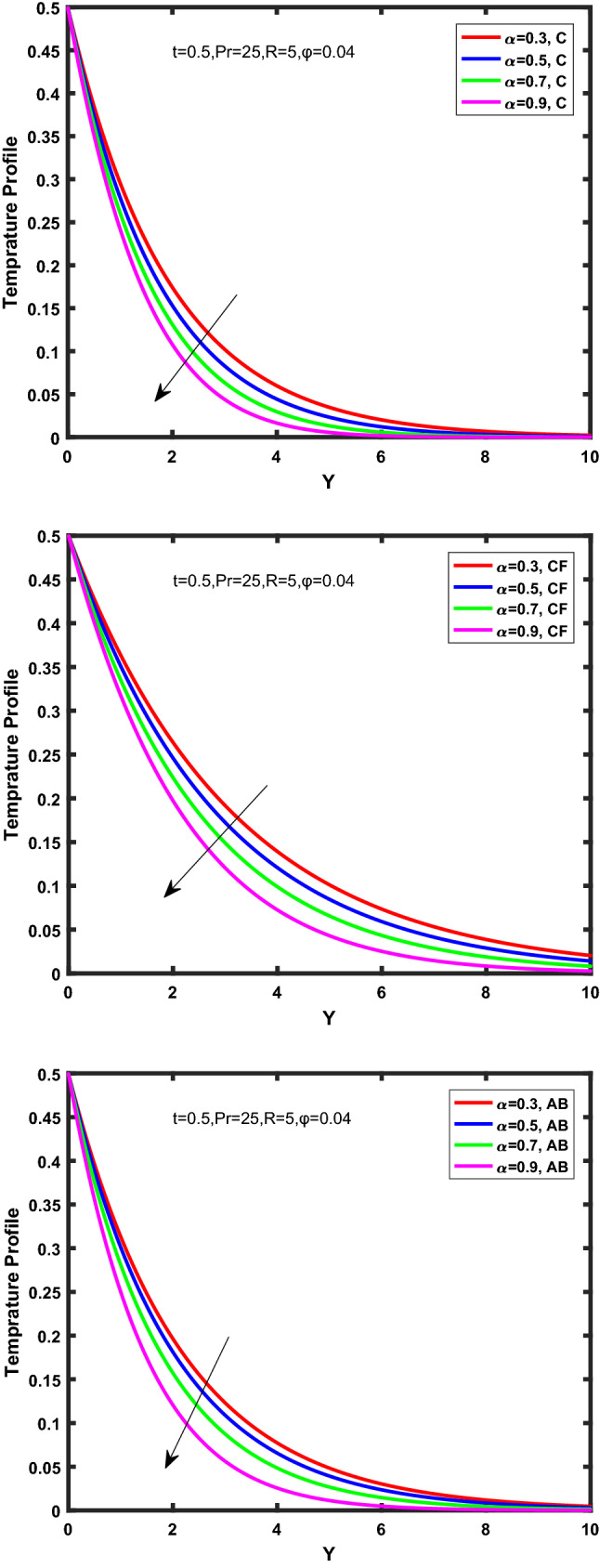
Temperature profile of Caputo, Caputo-Fabrizio, and Atangana-Baleanu for fractional parameter $\tilde{\alpha }$.

**Figure 5:**
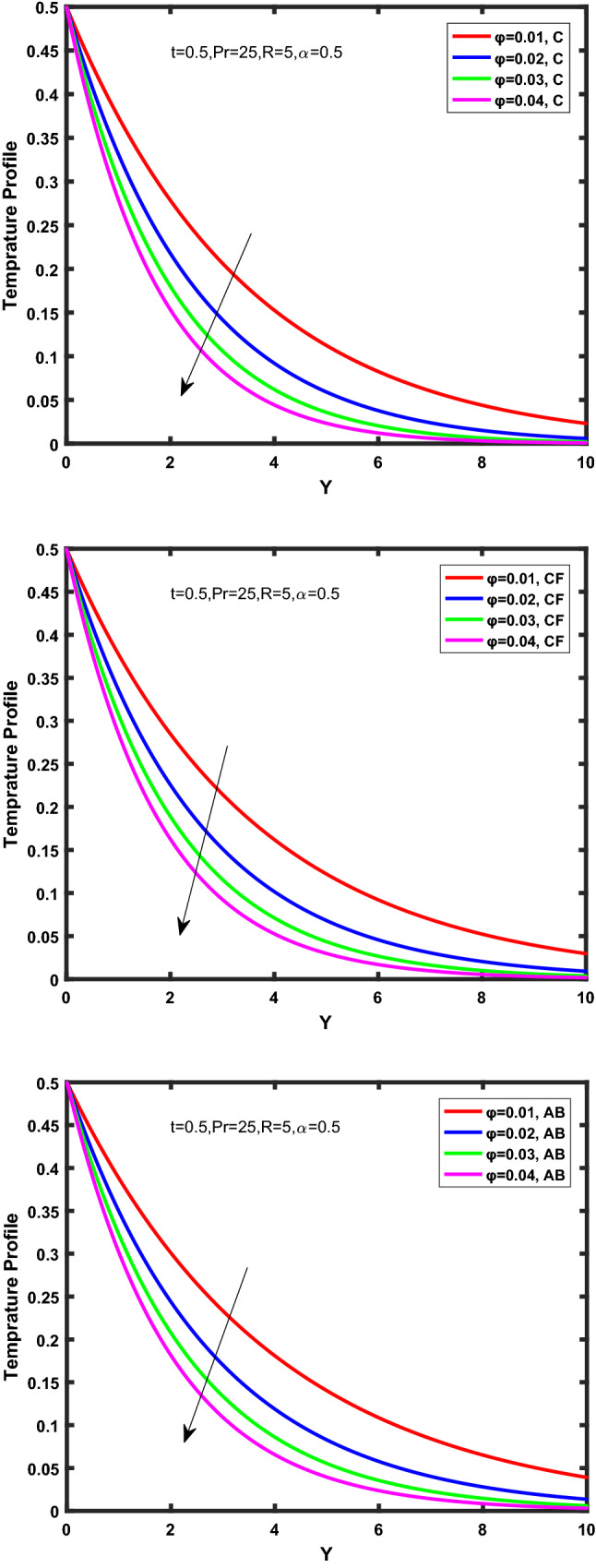
Temperature profile of Caputo, Caputo-Fabrizio, and Atangana-Baleanu for volume concentration $\ddot{\varphi }$.

**Figure 6:**
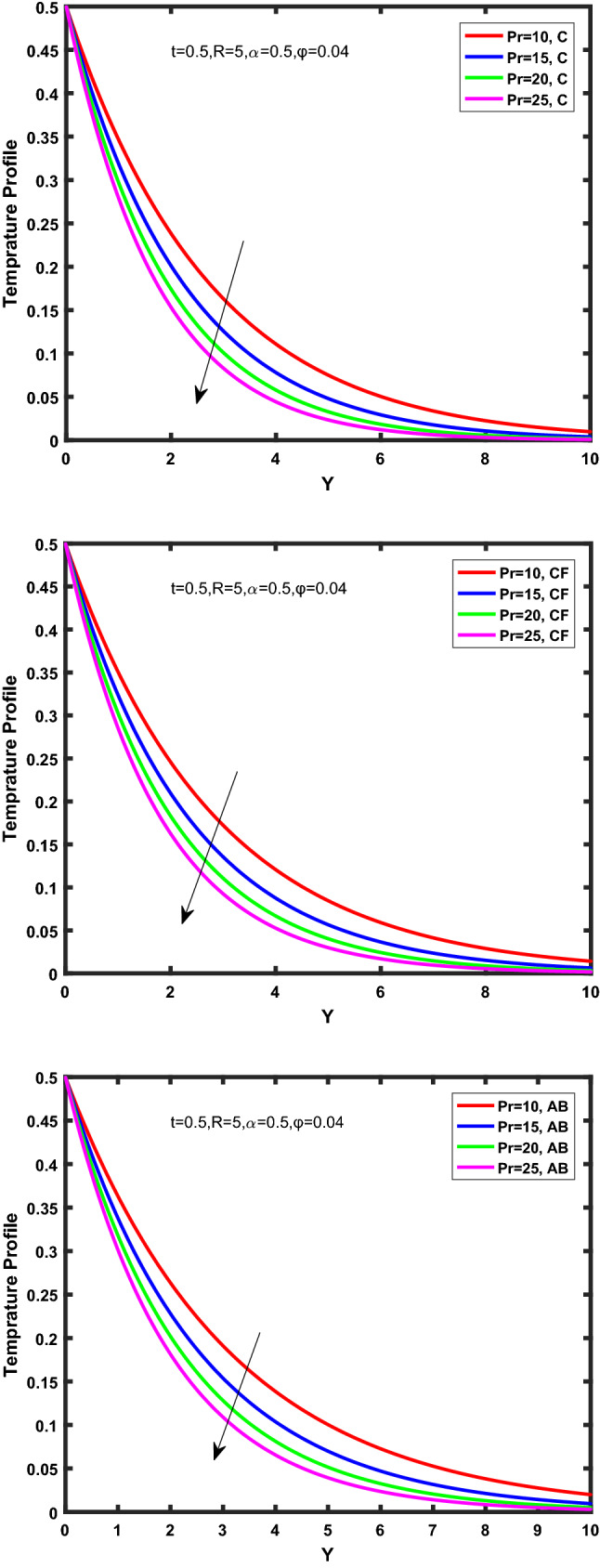
Temperature profile of Caputo, Caputo-Fabrizio, and Atangana-Baleanu for Prandtl number $\Pr$.

**Figure 7:**
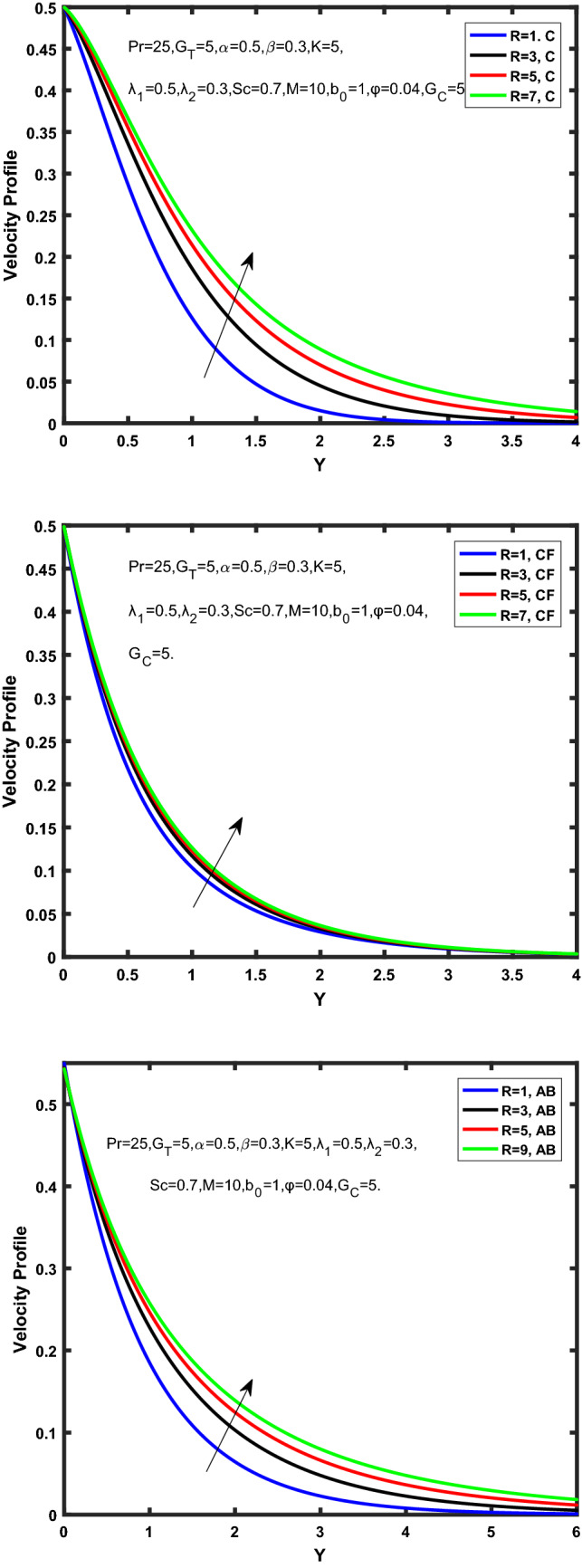
Velocity profile of Caputo, Caputo-Fabrizio, and Atangana-Baleanu for radiation factor,$\tilde{R}$.

**Figure 8:**
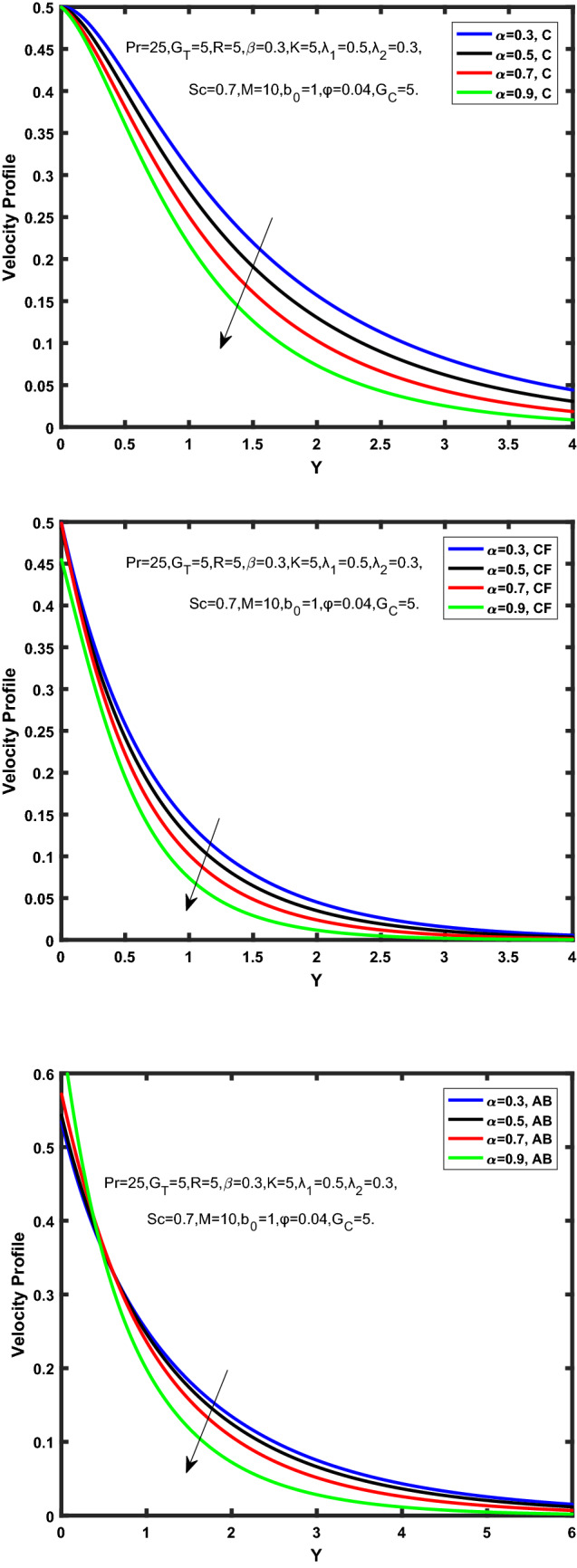
Velocity profile of Caputo, Caputo-Fabrizio, and Atangana-Baleanu for fractional factor, $\tilde{\alpha }$.

**Figure 9:**
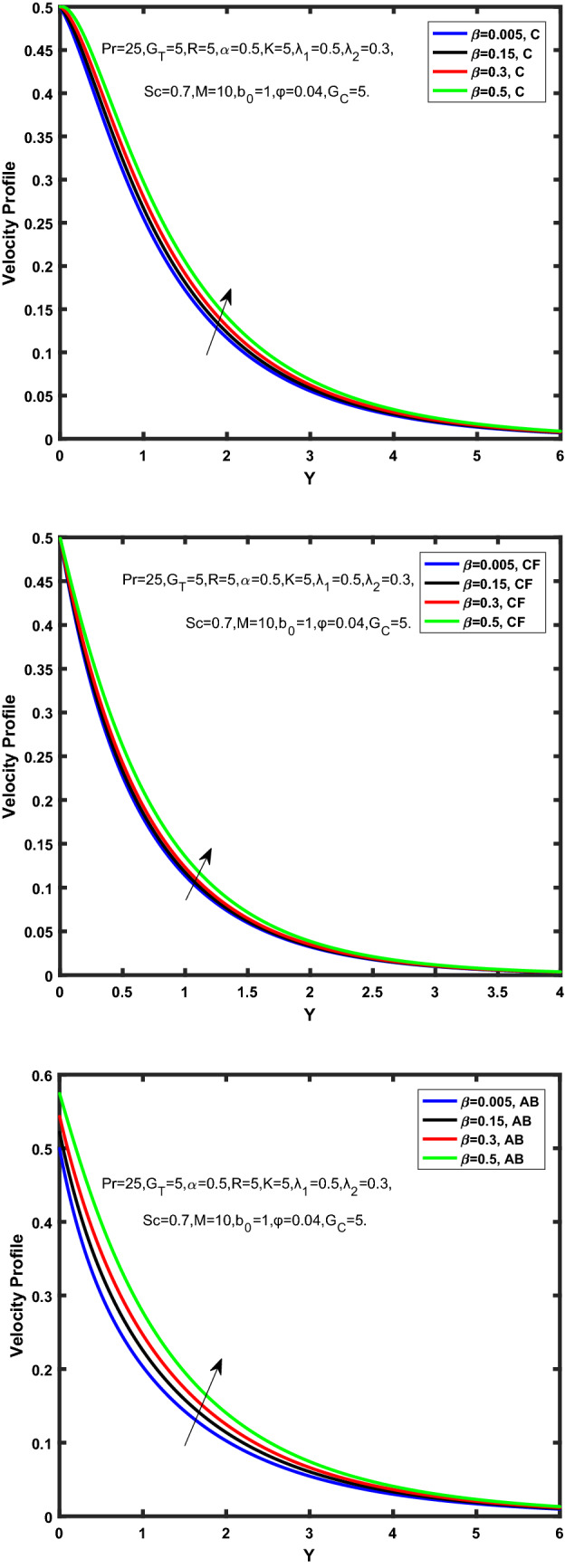
Velocity profile of Caputo, Caputo-Fabrizio, and Atangana-Baleanu for $\tilde{\beta }$.

**Figure 10:**
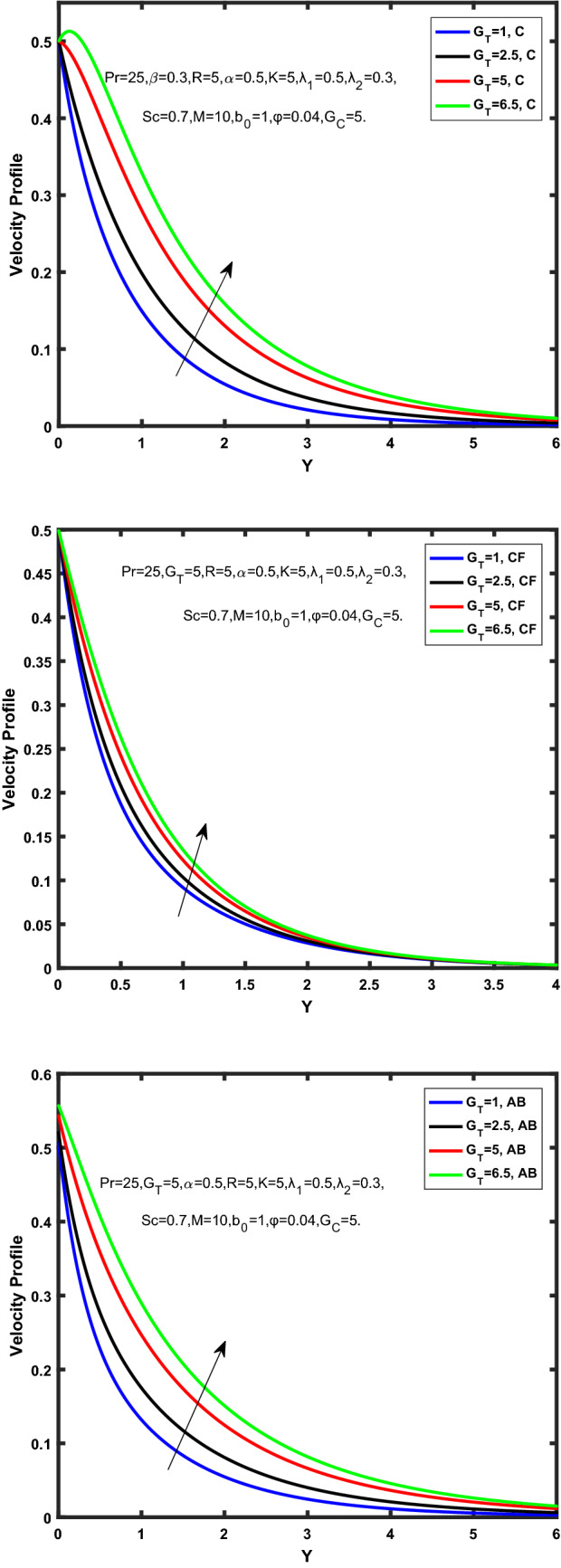
Velocity profile of Caputo, Caputo-Fabrizio, and Atangana-Baleanu for thermal Grashof number $\hat{G}_{T}$.

**Figure 11:**
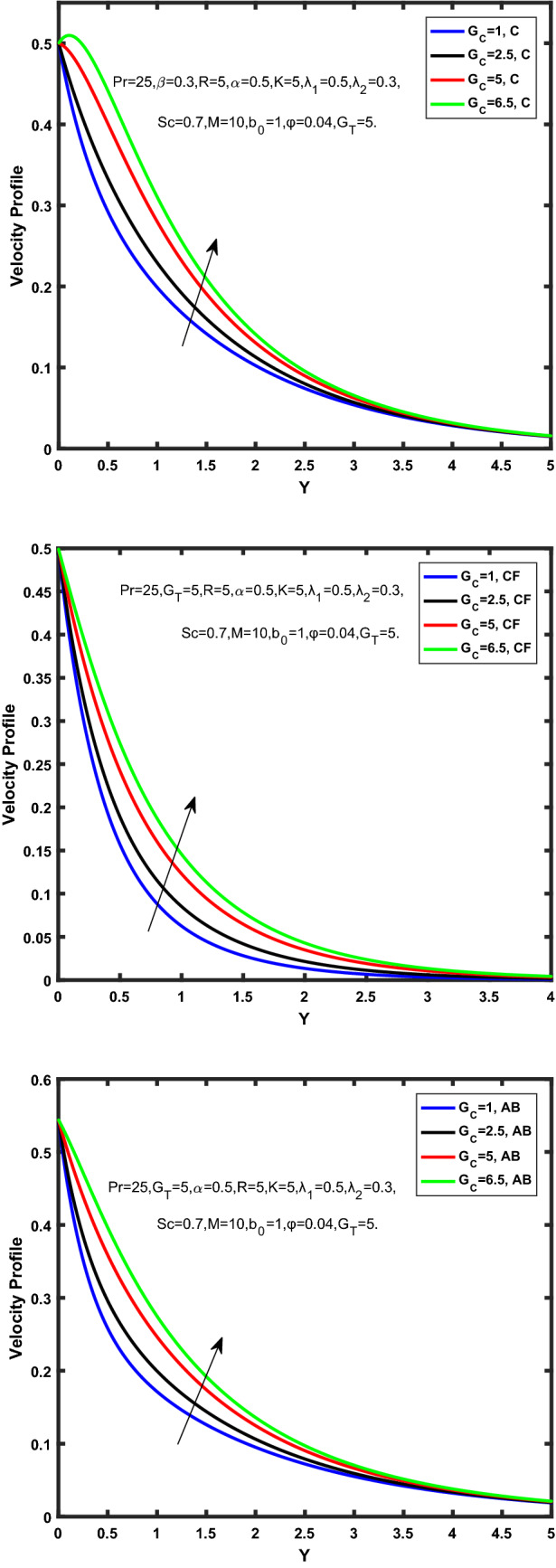
Velocity profile of Caputo, Caputo-Fabrizio, and Atangana-Baleanu for mass Grashof number, $\hat{G}_{C}$.

**Figure 12:**
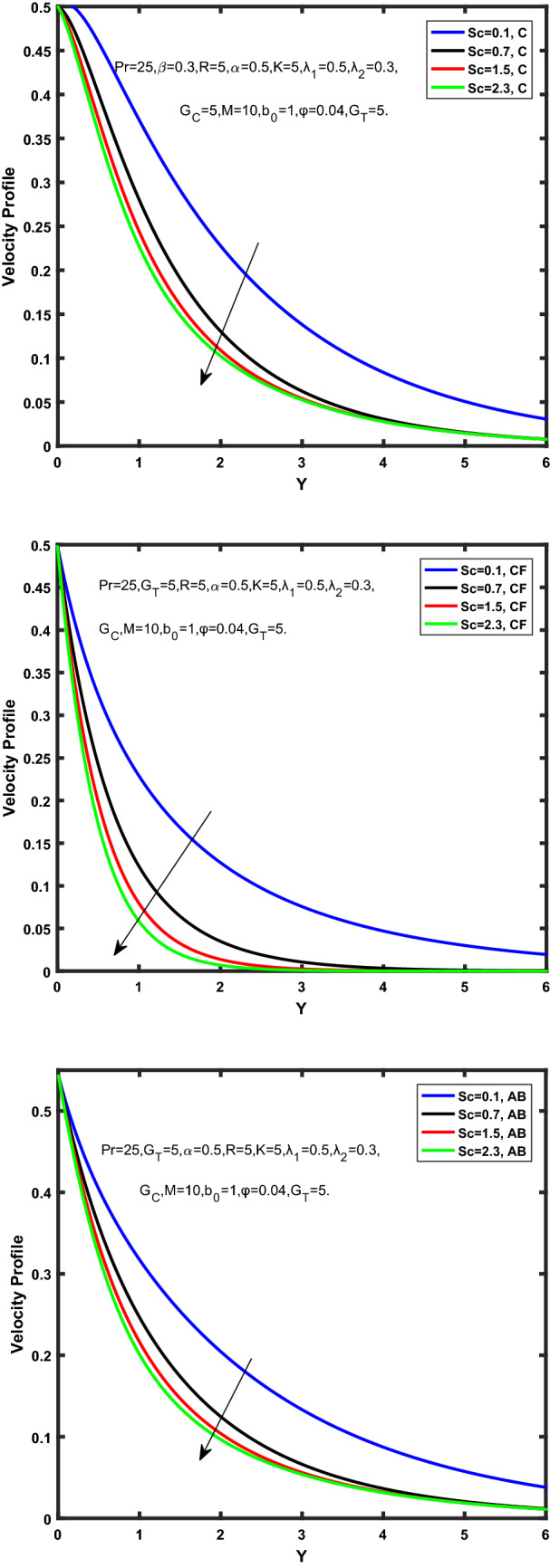
Velocity profile of Caputo, Caputo-Fabrizio, and Atangana-Baleanu for schmidt number, $Sc$.

**Figure 13:**
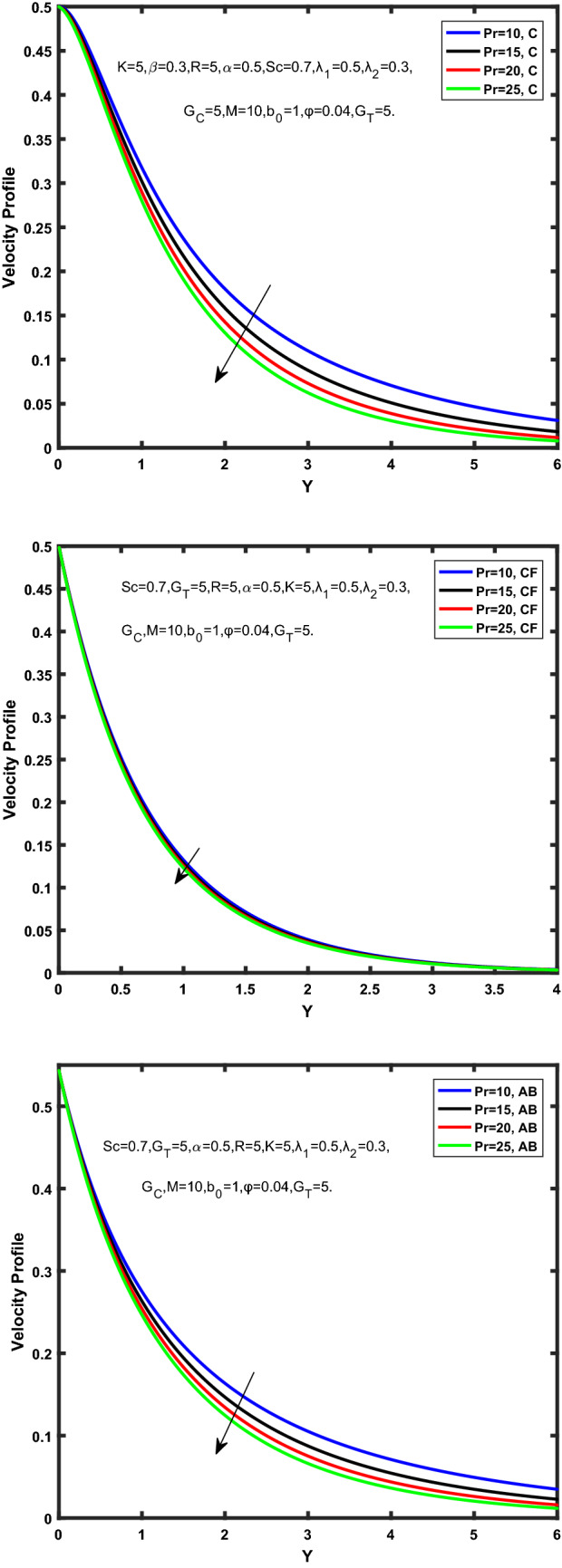
Velocity profile of Caputo, Caputo-Fabrizio, and Atangana-Baleanu for Prandtl number $\Pr$.

**Figure 14:**
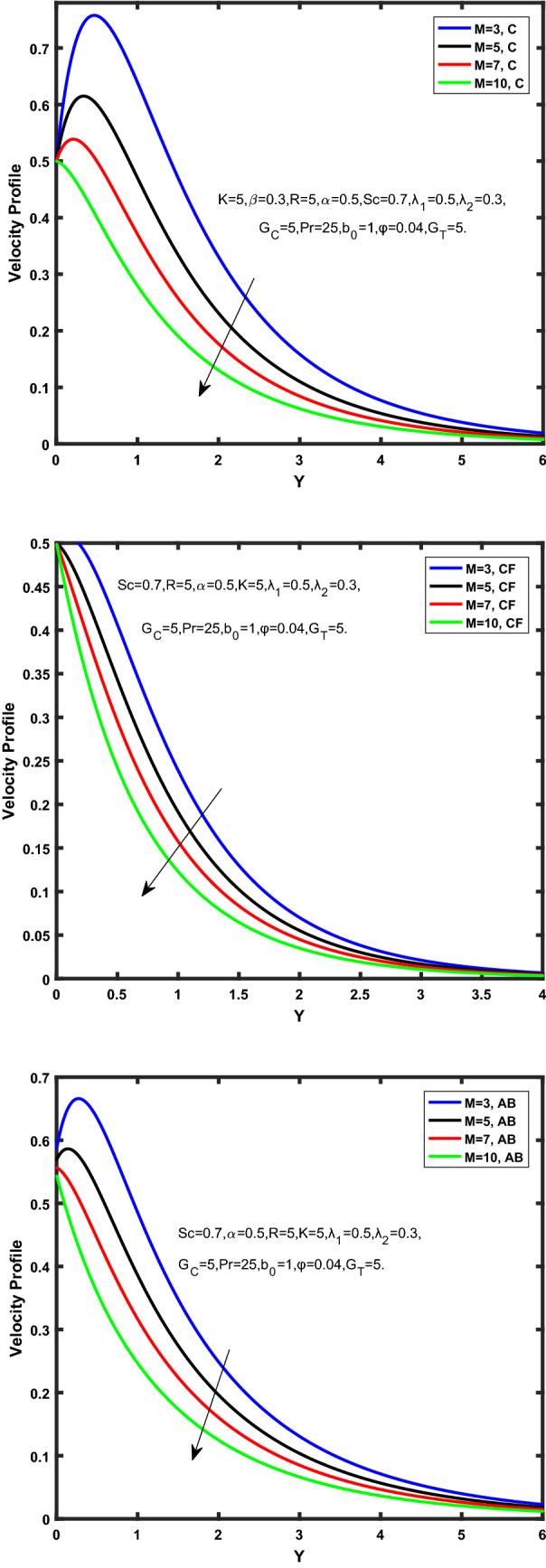
Velocity profile of Caputo, Caputo-Fabrizio, and Atangana-Baleanu for magnetic factor $\dddot M$.

**Figure 15:**
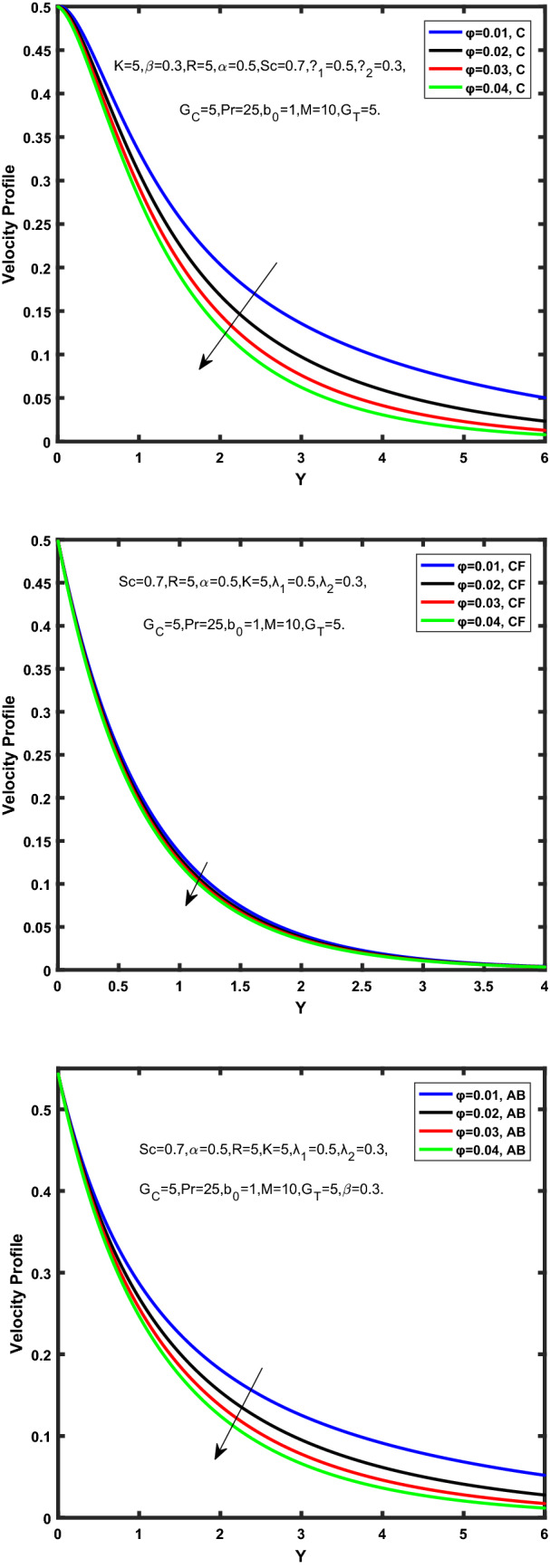
Velocity profile of Caputo, Caputo-Fabrizio, and Atangana-Baleanu for volume concentration $\ddot{\varphi }$.

**Figure 16:**
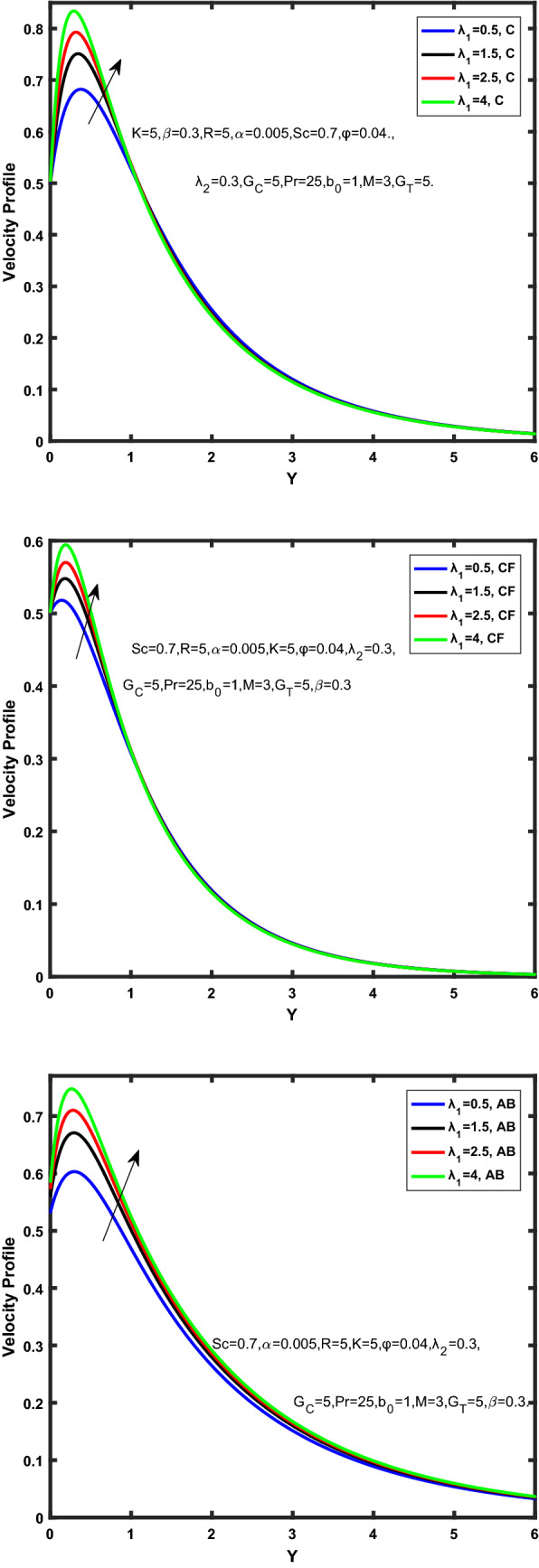
Velocity profile of Caputo, Caputo-Fabrizio, and Atangana-Baleanu for time relaxation $$\overset{\lower0.5em\hbox{$\smash{\scriptscriptstyle\frown}$}}{\overset{\lower0.5em\hbox{$\smash{\scriptscriptstyle\smile}$}}{\lambda } }_{1}$$.

**Figure 17:**
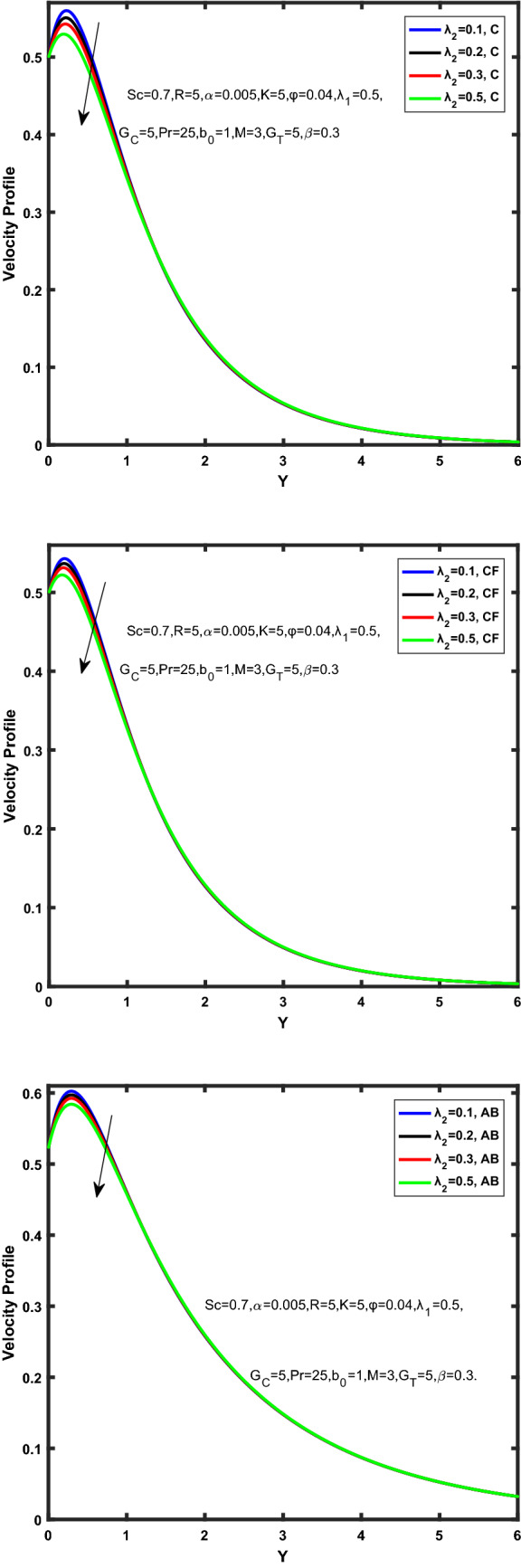
Velocity profile of Caputo, Caputo-Fabrizio, and Atangana-Baleanu for time retardation $$\overset{\lower0.5em\hbox{$\smash{\scriptscriptstyle\frown}$}}{\overset{\lower0.5em\hbox{$\smash{\scriptscriptstyle\smile}$}}{\lambda } }_{2}$$.

**Figure 18:**
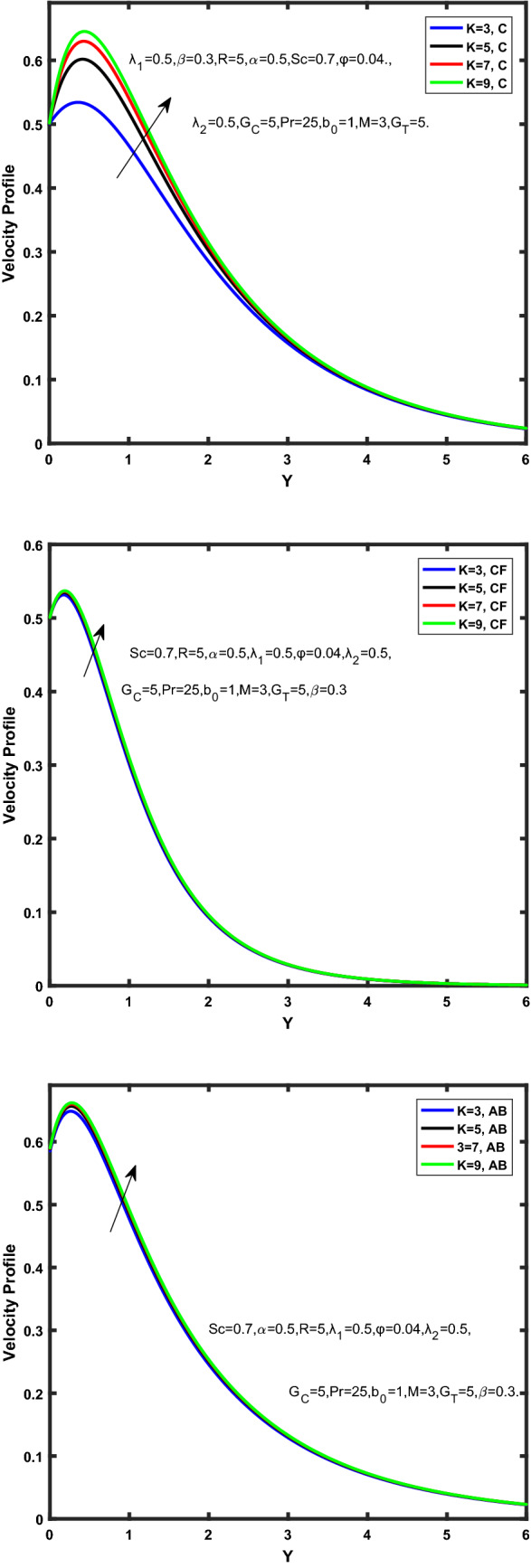
Velocity profile of Caputo, Caputo-Fabrizio, and Atangana-Baleanu for permeability of medium $$\overset{\lower0.5em\hbox{$\smash{\scriptscriptstyle\smile}$}}{K}$$.

Figure 3, 4, 5, 6 depict the temperature profile of Oldroyd-B nanofluid against different flow parameters. The impact of the radiation factor for various fractional models is shown in Fig. 3. Clearly, it illustrates that the temperature profile is accelerated for increasing value of radiation factor, $$\tilde{R}.$$ Since increase in $$\tilde{R}$$ at a fixed value of $$\overline{\tilde{T}}_{\infty }$$ and $$\overset{\lower0.5em\hbox{$\smash{\scriptscriptstyle\smile}$}}{k}_{nf}$$, reduces the value of $$\overline{\delta }^{ * }$$, therefore slope of radiative heat flux $$\frac{{\partial \tilde{q}_{r} }}{{\partial \overline{\tilde{Y}}}}$$ increases which pushes the radiative heat transfer rate to increase and gradually the fluid’s temperature rises. It shows that the thickness of energy boundary layer reduces and temperature is more uniformly distributed. Figure 4 presents the behavior of fractional parameter, $$\tilde{\alpha }$$ on the energy equation of nanofluid. It is witnessed that with the increase of $$\tilde{\alpha }$$, fall in temperature of Oldroyd-B nanofluid for all the fractional models is observed. The influence of the volume factor, $$\ddot{\varphi }$$ is presented in Fig. 5. It displays that the temperature for Caputo, CF, AB under the ramped conditions is decreased for the growing value of $$\ddot{\varphi }$$. Physically, it had to be happened that the rate of heat transmission must fall with increasing the volume factor of nanoparticles in the nanofluid. It is due to the growing resistance among the particles in nanofluid that resultantly reduced the flow of Oldroyd-B nanofluid. Figure 6 demonstrates the impact of the Prandtl number on the temperature profile of the fractional nanofluid. It highlights that elevating the Prandtl number, decreases the temperature field. The Prandtl number and thermal conductivity are inversely related. The reason behind this is that as we enhance the Prandtl number, it reduces the thermal conductivity, which in result reduces both the thermal boundary layer and conductance thickness. So as a result, the nanofluid is subjected to higher thermal tolerance and temperature decay. Figures 7, 8, 9, 10, 11, 12, 13, 14, 15, 16, 17, 18 present the velocity profile of Oldroyd B- nanofluid under different circumstances.

Figure 7 represents the behavior of the radiation factor $\tilde{R}$ on the velocity of the nanofluid. It is found that the enlargement of the radiation factor $\tilde{R}$ boosts the velocity of fractional nanofluid models. This is due to growing the value of $\tilde{R}$ escalating the rate of heat transmission. Because of this accelerating rate of the heat transfer makes the bonding among the particles of nanofluid weak and eventually this reduces the interaction of the viscous forces. The weak viscous forces give freedom to the nanofluid to flow with higher velocity. The impact of $\tilde{\alpha }$ on the velocity profile is presented in Fig. 8. It displays that the velocity of nanofluid depletes with the elevation of the value of fractional parameter $\tilde{\alpha }$. Physically, there is an inverse relation between $\tilde{\alpha }$ and momentum boundary layer thickness under the ramped conditions. However, when $\tilde{\alpha }$ accelerates, the momentum boundary layer reduces which results in the reduction the velocity profile. Figure 9 shows the effect of $\tilde{\beta }$, which illustrates that the velocity profile aggrandizes with an increase of $\tilde{\beta }$. Figure 10 depicts the influence of thermal Grashof number $\hat{G}_{T}$. Clearly, the velocity of nanofluid boosts with enhancing the value of $\hat{G}_{T}$ in case of fractional models. Physically, this characteristic is due to increase of the viscous and thermal forces during the flow. The larger the value of $\hat{G}_{T}$ depicting the nanofluid is energized that bolsters the impact of thermal force because of the existence of convection current. These currents get the value of great importance due to prevailing temperature slop and eventually cause the viscous forces to sink. As a result, the nanofluid velocity enhances and we obtained the elevated velocity profile. From Fig. 11 we see that the velocity of nanofluid increases with the growing value of mass Grashof number $\hat{G}_{C}$ under ramped conditions. This is because of viscous and mass buoyancy forces. The impact of the Schmidt number $Sc$ is displayed in Fig. 12. It highlights the reduction of velocity profile with increasing value of $Sc.$ An escalation in $Sc$ gives the domination of viscous force on the diffusional impact. Schmidt number in fact gives the relative effectiveness of mass and momentum diffusion in specie (concentration) and momentum boundary layers. Lower values of $Sc$ associates to smaller molecular weight species or diffusing while greater values to a denser diffusing medium. Therefore, growing value of $Sc$ has an inverse effect on momentum diffusion, since the viscosity of nanofluid will higher and molecular diffusivity will be lowered. That’s why the flow of nanofluid is decreasing as we increase the value of $Sc.$

**Figure 19:**
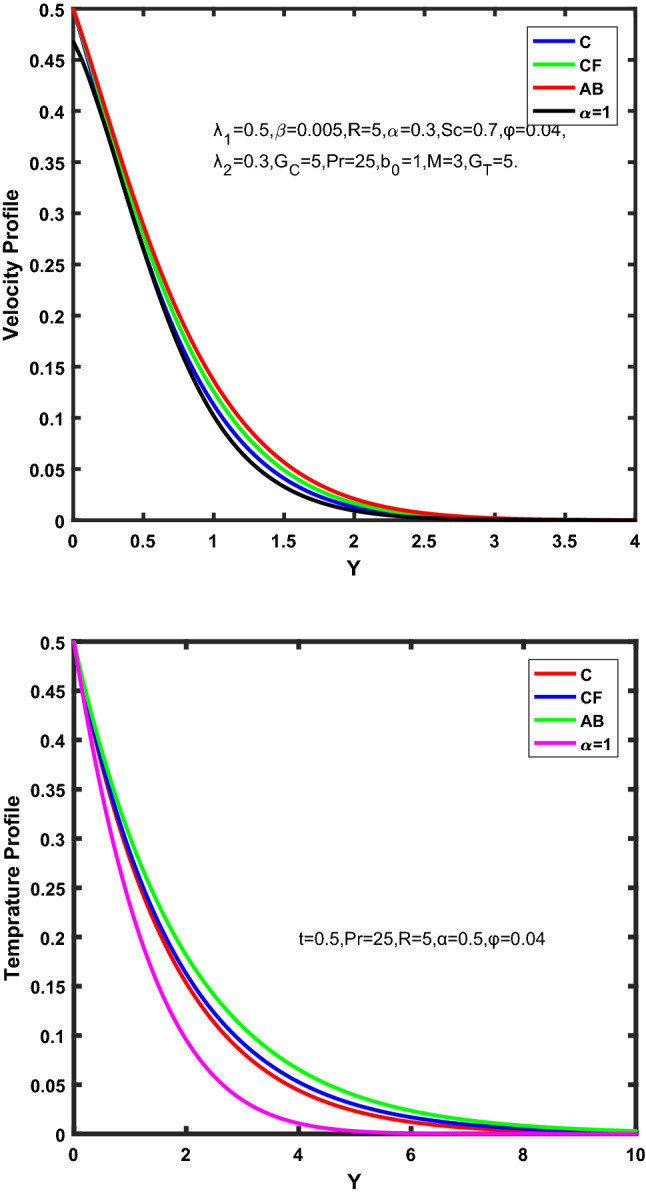
Comparison of velocity and temperature Profile of Caputo, Caputo-Fabrizio, Atangana-Baleanu, and ordinary fluid.

**Figure 20:**
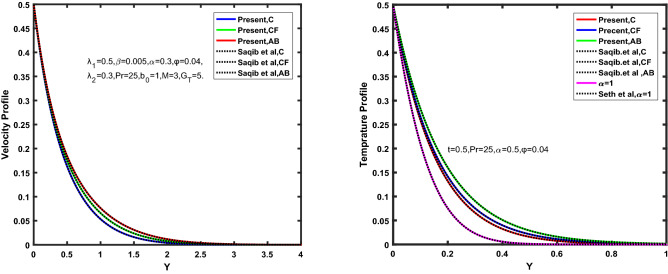
Validation of temperature and velocity fields for Caputo, Caputo-Fabrizio, Atangana-Baleanu.

Figure 13 displays the physical behavior of Prandtl number Pr. The Prandtl number is the ratio of momentum diffusivity (kinematic viscosity) to thermal diffusivity of a fluid. It shows that the speed of nanofluid flow de-escalates with the escalating value of Pr with ramped conditions. The Pr of blood is taken as 25. Physically, for the greater value of Pr, the thermal conductivity of nanofluid is smaller due to increase in viscosity which further causes the reduction in the velocity of nanofluid. The influence of the magnetic parameter on momentum limiting layer is shown in Fig. 14. It is found that if we boost the value of the magnetic factor $$\dddot M$$, the nanofluid decelerates. This is due to Lorentz’s force which causes resistance in the motion that occurs because of the transverse applied magnetic field, which is responsible for the falling of velocity of nanofluid. Figure 15 depicts the effect of $$\ddot{\varphi }$$ on the velocity field. It shows if we enhance the $$\ddot{\varphi }$$, the velocity of nanofluid is reduced for ramped condition. There is a direct relation between the viscosity and $$\ddot{\varphi }$$, if we grow the value of volume concentration it boosts the viscosity of nanofluid and it becomes thick. Figures 16, 17 characterize the behavior of relaxation and retardation time on fluid’s velocity. It represents that the nanofluid’s velocity escalates with incrementing the value of $$\overset{\lower0.5em\hbox{$\smash{\scriptscriptstyle\frown}$}}{\overset{\lower0.5em\hbox{$\smash{\scriptscriptstyle\smile}$}}{\lambda } }_{1}$$, while $$\overset{\lower0.5em\hbox{$\smash{\scriptscriptstyle\frown}$}}{\overset{\lower0.5em\hbox{$\smash{\scriptscriptstyle\smile}$}}{\lambda } }_{2}$$ shows inverse effect, the motion of the fluid reduces with increasing $$\overset{\lower0.5em\hbox{$\smash{\scriptscriptstyle\frown}$}}{\overset{\lower0.5em\hbox{$\smash{\scriptscriptstyle\smile}$}}{\lambda } }_{2}$$. From this, we conclude that, if we increase $$\overset{\lower0.5em\hbox{$\smash{\scriptscriptstyle\frown}$}}{\overset{\lower0.5em\hbox{$\smash{\scriptscriptstyle\smile}$}}{\lambda } }_{1}$$, fluid will take a smaller time to come in the rest position. It is found in Fig. 18 that increase in the permeability of medium boosts the boundary layer thickness of the fluid. The escalation in permeability reduces the tolerance which in turn accelerates the momentum development of the regime that’s why the nanofluid’s velocity increases. Figure 19 investigates the comparison of various fractional time derivative (C, CF, AB) and integer-order derivative $$\left( {\tilde{\alpha } \to 1} \right)$$ of both velocity and temperature profiles. In order to authenticate our obtained solutions, a comparative analysis is presented in Fig. 20. It can be observed if the radiation parameter $$\tilde{R}$$ is removed from temperature field and $$\tilde{R}{\text{ and }}\hat{G}_{C}^{1}$$ are deleted from the velocity field of the current model, then the present solutions for temperature and velocity field are in excellent agreement with velocity and temperature solutions of^21^. Similarly, if $$\tilde{\alpha } \to 1$$ and $$\tilde{R}$$ are removed from temperature field of present solution, then we will obtain the solution of^55^. Tables 2, 3 highlight the computations of dimensionless governing equations of Caputo, CF, and AB by Zakian’s and Stehfest’s algorithms at various spatial steps to confirm the authenticity of our solutions up to the desired accuracy. Additionally, it also depicts AB fractional model has higher values as compared to the other’s fractional models. It can be seen easily that AB fractional derivative better explains the memory impact of the solutions of temperature, concentration, and velocity profiles as compared to Caputo and Caputo-Fabrizio. As the kernel of Atangana-Baleanu possesses the characteristics of without singularity and locality because of this it is more efficient than the Caputo and Caputo-Fabrizio. It also displays that the concentration of the nanofluid reduces with the increasing value of Schmidt number. It also describes that the temperature and velocity both increase with increasing the increasing values of $$\tilde{R},\tilde{\beta },\hat{G}_{T} ,\hat{G}_{C} ,\overset{\lower0.5em\hbox{$\smash{\scriptscriptstyle\frown}$}}{\overset{\lower0.5em\hbox{$\smash{\scriptscriptstyle\smile}$}}{\lambda } }_{1} ,\overset{\lower0.5em\hbox{$\smash{\scriptscriptstyle\smile}$}}{K}$$, while decrease for the rest of parameters.

**Table 2:** Represent the solutions of the flow problem utilizing Zakian’s algorithms with $$\hat{G}_{T} = 5,\tilde{R} = 5,\ddot{\varphi } = 0.04,\hat{G}_{C} ,Sc = 0.7,\dddot M = 10,Pr = 25,\overset{\lower0.5em\hbox{$\smash{\scriptscriptstyle\frown}$}}{\overset{\lower0.5em\hbox{$\smash{\scriptscriptstyle\smile}$}}{\lambda } }_{1} = 0.5,\overset{\lower0.5em\hbox{$\smash{\scriptscriptstyle\frown}$}}{\overset{\lower0.5em\hbox{$\smash{\scriptscriptstyle\smile}$}}{\lambda } }_{2} = 0.3,\overset{\lower0.5em\hbox{$\smash{\scriptscriptstyle\smile}$}}{K} = 5,\tilde{\alpha } = 0.5,\tilde{\beta } = 0.3.$$

$$\tilde{\alpha }$$	$$\tilde{\beta }$$	$$\tilde{R}$$	$$\ddot{\varphi }$$	Zakian’s $$\tilde{\Phi }$$ (C)	Zakian’s $$\tilde{\Phi }$$ (CF)	Zakian’s $$\tilde{\Phi }$$ (AB)	Zakian’s $$\overset{\lower0.5em\hbox{$\smash{\scriptscriptstyle\frown}$}}{\Theta }$$(C)	Zakian’s $$\overset{\lower0.5em\hbox{$\smash{\scriptscriptstyle\frown}$}}{\Theta }$$(CF)	Zakian’s $$\overset{\lower0.5em\hbox{$\smash{\scriptscriptstyle\frown}$}}{\Theta }$$(AB)	Zakian’s $$\tilde{U}^{ * }$$(C)	Zakian’s $$\tilde{U}^{ * }$$(CF)	Zakian’s $$\tilde{U}^{ * }$$(AB)
0.5	0.3	5	.04	0.11289	0.12274	0.14066	0.02837	0.03552	0.04593	0.06086	0.06288	0.07411
0.9	0.3	5	.04	0.06964	0.07421	0.08277	0.00781	0.01150	0.01454	0.02787	0.03087	0.03539
0.5	0.3	5	.04	-	-	-	-	-	-	0.06086	0.06288	0.07411
0.5	0.5	5	.04	-	-	-	-	-	-	0.06097	0.06298	0.07418
0.5	0.3	3	.04	0.07235	0.08183	0.09756	-	-	-	0.04394	0.04694	0.05730
0.5	0.3	5	.04	0.11289	0.12274	0.14066	-	-	-	0.06086	0.06288	0.07411
0.5	0.3	5	.02	0.17587	0.18500	0.20372	-	-	-	0.08719	0.08722	0.09879
0.5	0.3	5	.04	0.11289	0.12274	0.14066	-	-	-	0.06086	0.06288	0.07411

$$\Pr$$	$$Sc$$	$$\overset{\lower0.5em\hbox{$\smash{\scriptscriptstyle\frown}$}}{\overset{\lower0.5em\hbox{$\smash{\scriptscriptstyle\smile}$}}{\lambda } }_{1}$$	$$\overset{\lower0.5em\hbox{$\smash{\scriptscriptstyle\frown}$}}{\overset{\lower0.5em\hbox{$\smash{\scriptscriptstyle\smile}$}}{\lambda } }_{2}$$	Zakian’s $$\tilde{\Phi }$$ (C)	Zakian’s $$\tilde{\Phi }$$ (CF)	Zakian’s $$\tilde{\Phi }$$ (AB)	Zakian’s $$\overset{\lower0.5em\hbox{$\smash{\scriptscriptstyle\frown}$}}{\Theta }$$ (C)	Zakian’s $$\overset{\lower0.5em\hbox{$\smash{\scriptscriptstyle\frown}$}}{\Theta }$$ (CF)	Zakian’s $$\overset{\lower0.5em\hbox{$\smash{\scriptscriptstyle\frown}$}}{\Theta }$$(AB)	Zakian’s $$\tilde{U}^{ * }$$ (C)	Zakian’s $$\tilde{U}^{ * }$$(CF)	Zakian’s $$\tilde{U}^{ * }$$(AB)
10	0.3	0.5	0.3	0.19718	0.20588	0.22437	-	-	-	0.09612	0.09542	0.10691
25	0.3	0.5	0.3	0.11289	0.12274	0.14066	-	-	-	0.06086	0.06288	0.07411
25	0.3	0.5	0.3	-	-	-	0.07918	0.08880	0.10503	0.08217	0.08364	0.09714
25	0.7	0.5	0.3	-	-	-	0.02837	0.03552	0.04593	0.06086	0.06288	0.07411
25	0.3	0.5	0.3	-	-	-	-	-	-	0.06086	0.06288	0.07411
25	0.3	4	0.3	-	-	-	-	-	-	0.05962	0.06198	0.07330
25	0.3	0.5	0.3	-	-	-	-	-	-	0.06086	0.06288	0.07411
25	0.3	0.5	0.7	-	-	-	-	-	-	0.06155	0.06337	0.07451

$$\hat{G}_{T}$$	$$\hat{G}_{C}$$	$$\dddot M$$	$$\overset{\lower0.5em\hbox{$\smash{\scriptscriptstyle\smile}$}}{K}$$	Zakian’s $$\tilde{U}^{ * }$$ (C)	Zakian’s $$\tilde{U}^{ * }$$(CF)	Zakian’s $$\tilde{U}^{ * }$$(AB)						
1	5	10	5	0.02237	0.02414	0.02970						
5	5	10	5	0.06086	0.06288	0.07411						
5	1	10	5	0.05067	0.05132	0.05924						
5	6.5	10	5	0.06468	0.06721	0.07969						
5	5	3	5	0.15116	0.12542	0.14741						
5	5	10	5	0.06086	0.06288	0.07411						
5	5	10	3	0.06036	0.06240	0.07353						
5	5	10	5	0.06086	0.06288	0.07411

**Table 3:** Represent the solutions of the flow problem utilizing Stehfest’s algorithms with $$\hat{G}_{T} = 5,\tilde{R} = 5,\ddot{\varphi } = 0.04,\hat{G}_{C} ,Sc = 0.7,\dddot M = 10,Pr = 25,\overset{\lower0.5em\hbox{$\smash{\scriptscriptstyle\frown}$}}{\overset{\lower0.5em\hbox{$\smash{\scriptscriptstyle\smile}$}}{\lambda } }_{1} = 0.5,\overset{\lower0.5em\hbox{$\smash{\scriptscriptstyle\frown}$}}{\overset{\lower0.5em\hbox{$\smash{\scriptscriptstyle\smile}$}}{\lambda } }_{2} = 0.3,\overset{\lower0.5em\hbox{$\smash{\scriptscriptstyle\smile}$}}{K} = 5,\tilde{\alpha } = 0.5,\tilde{\beta } = 0.3.$$

$$\tilde{\alpha }$$	$$\tilde{\beta }$$	$$\tilde{R}$$	$$\ddot{\varphi }$$	Stehfest’s $$\tilde{\Phi }$$ (C)	Stehfest’s $$\tilde{\Phi }$$ (CF)	Stehfest’s $$\tilde{\Phi }$$ (AB)	Stehfest’s $$\overset{\lower0.5em\hbox{$\smash{\scriptscriptstyle\frown}$}}{\Theta }$$(C)	Stehfest’s $$\overset{\lower0.5em\hbox{$\smash{\scriptscriptstyle\frown}$}}{\Theta }$$(CF)	Stehfest’s $$\overset{\lower0.5em\hbox{$\smash{\scriptscriptstyle\frown}$}}{\Theta }$$(AB)	Stehfest’s $$\tilde{U}^{ * }$$(C)	Stehfest’s $$\tilde{U}^{ * }$$(CF)	Stehfest’s $$\tilde{U}^{ * }$$(AB)
0.5	0.3	5	.04	0.11320	0.12310	0.14109	0.02842	0.03561	0.04606	0.06093	0.06291	0.07416
0.9	0.3	5	.04	0.06970	0.07434	0.08292	0.00779	0.01151	0.01455	0.02801	0.03097	0.03550
0.5	0.3	5	.04	-	-	-	-	-	-	0.06093	0.06291	0.07416
0.5	0.5	5	.04	-	-	-	-	-	-	0.06104	0.06300	0.07422
0.5	0.3	3	.04	0.07253	0.08206	0.09785	-	-	-	0.04402	0.04697	0.05735
0.5	0.3	5	.04	0.11320	0.12310	0.14109	-	-	-	0.06093	0.06291	0.07416
0.5	0.3	5	.02	0.17640	0.18555	0.20435	-	-	-	0.08720	0.08724	0.09884
0.5	0.3	5	.04	0.11320	0.12310	0.14109	-	-	-	0.06093	0.06291	0.07416

$$\Pr$$	$$Sc$$	$$\overset{\lower0.5em\hbox{$\smash{\scriptscriptstyle\frown}$}}{\overset{\lower0.5em\hbox{$\smash{\scriptscriptstyle\smile}$}}{\lambda } }_{1}$$	$$\overset{\lower0.5em\hbox{$\smash{\scriptscriptstyle\frown}$}}{\overset{\lower0.5em\hbox{$\smash{\scriptscriptstyle\smile}$}}{\lambda } }_{2}$$	Stehfest’s $$\tilde{\Phi }$$ (C)	Stehfest’s $$\tilde{\Phi }$$ (CF)	Stehfest’s $$\tilde{\Phi }$$ (AB)	Stehfest’s $$\overset{\lower0.5em\hbox{$\smash{\scriptscriptstyle\frown}$}}{\Theta }$$(C)	Stehfest’s $$\overset{\lower0.5em\hbox{$\smash{\scriptscriptstyle\frown}$}}{\Theta }$$(CF)	Stehfest’s $$\overset{\lower0.5em\hbox{$\smash{\scriptscriptstyle\frown}$}}{\Theta }$$(AB)	Stehfest’s $$\tilde{U}^{ * }$$(C)	Stehfest’s $$\tilde{U}^{ * }$$(CF)	Stehfest’s $$\tilde{U}^{ * }$$(AB)
10	0.3	0.5	0.3	0.19779	0.20649	0.22506	-	-	-	0.09611	0.09544	0.10694
25	0.3	0.5	0.3	0.11320	0.12310	0.14109	-	-	-	0.06093	0.06291	0.07416
25	0.3	0.5	0.3	-	-	-	0.07938	0.08906	0.10534	0.08225	0.08367	0.09719
25	0.7	0.5	0.3	-	-	-	0.02842	0.03561	0.04606	0.06093	0.06291	0.07416
25	0.3	0.5	0.3	-	-	-	-	-	-	0.06093	0.06291	0.07416
25	0.3	4	0.3	-	-	-	-	-	-	0.05969	0.06200	0.07335
25	0.3	0.5	0.3	-	-	-	-	-	-	0.06093	0.06291	0.07416
25	0.3	0.5	0.7	-	-	-	-	-	-	0.06161	0.06339	0.07456

$$\hat{G}_{T}$$	$$\hat{G}_{C}$$	$$\dddot M$$	$$\overset{\lower0.5em\hbox{$\smash{\scriptscriptstyle\smile}$}}{K}$$	Stehfest’s $$\tilde{U}^{ * }$$(C)	Stehfest’s $$\tilde{U}^{ * }$$(CF)	Stehfest’s $$\tilde{U}^{ * }$$(AB)						
1	5	10	5	0.02242	0.02415	0.02973						
5	5	10	5	0.06093	0.06291	0.07416						
5	1	10	5	0.05071	0.05134	0.05927						
5	5	10	5	0.06093	0.06291	0.07416						
5	5	3	5	0.15145	0.12570	0.14778						
5	5	10	5	0.06093	0.06291	0.07416						
5	5	10	3	0.06043	0.06243	0.07357						
5	5	10	5	0.06093	0.06291	0.07416						

## Conclusion

This aims to study the heat and mass transfer of Oldroyd-B bio-magnetic nanofluid with ramped conditions in the permeable medium. Some unitless parameters are used to make the dimensional governing equations into non-dimensional form. To develop the resulting Oldroyd-B nanofluid model three type definitions of fractional operators are introduced. LT technique is used for the solutions of the flow problem. For comparison and authenticity of our solutions, Zakian’s and Stehfest’s algorithms are used. Following key points are concluded from this investigation;

- The temperature profile of nanofluid increases with incrementing the value of $\tilde{R}$, It means that the thickness of energy boundary layer decreases and temperature is more uniformly distributed, while reduces it reduces with increase in $\tilde{\alpha },\ddot{\varphi },{\text{ and }}\Pr ,$ because the rate of heat transfer decreases with increasing $\ddot{\varphi }$, this is due to the higher value viscosity of nanofluid reduces the flow of fluid. Also, with increasing Prandtl number $\Pr$ decreases the temperature of nanofluid, because the thermal conductivity reduces, which further cause reduction to both the thickness of the thermal boundary layer and conductance.
- The velocity of nanofluid de-escalates with the increase of volume concentration $\ddot{\varphi }.$ However, the viscosity and the volume concentration have a direct relation. If we increase the value of $\ddot{\varphi }$ that increases the viscosity of nanofluid and it becomes thick. Because of this the velocity of nanofluid decreases.
- Concentration and velocity profiles get reduced while accelerating the Schmidt number $Sc.$ An increasing value of Schmidt number gives the domination of viscous force on the diffusional effect. Due to this flow of nanofluid slows down.
- The fluid’s velocity boosts with various parameters like $$\hat{G}_{T} ,\tilde{\beta },\hat{G}_{C} ,\tilde{R},{\text{ and }}\overset{\lower0.5em\hbox{$\smash{\scriptscriptstyle\smile}$}}{K}$$ for Caputo, CF, and AB fractional models.
- For the growing value of $\dddot M,\,\Pr {\text{ and }}\tilde{\alpha }$, the velocity profile reduces.
- The velocity of nanofluid rises near the plate with increasing $$\overset{\lower0.5em\hbox{$\smash{\scriptscriptstyle\frown}$}}{\overset{\lower0.5em\hbox{$\smash{\scriptscriptstyle\smile}$}}{\lambda } }_{1}$$ but depletes after some critical value of $$\overline{\tilde{Y}}$$, while $$\overset{\lower0.5em\hbox{$\smash{\scriptscriptstyle\frown}$}}{\overset{\lower0.5em\hbox{$\smash{\scriptscriptstyle\smile}$}}{\lambda } }_{2}$$ shows the inverse effect to that of $$\overset{\lower0.5em\hbox{$\smash{\scriptscriptstyle\frown}$}}{\overset{\lower0.5em\hbox{$\smash{\scriptscriptstyle\smile}$}}{\lambda } }_{1}$$.

## Nomenclature

$$\overset{\lower0.5em\hbox{$\smash{\scriptscriptstyle\frown}$}}{\tilde{\rho }}_{nf}$$: Density of nanofluid
$$\overset{\lower0.5em\hbox{$\smash{\scriptscriptstyle\frown}$}}{\tilde{\lambda }}_{m}$$: Relaxation time
$$\overset{\lower0.5em\hbox{$\smash{\scriptscriptstyle\frown}$}}{\tilde{\mu }}_{nf}$$: Absolute viscosity of nanofluid
$B_{0}^{2}$: Magnetic field strength
$$\overset{\lower0.5em\hbox{$\smash{\scriptscriptstyle\frown}$}}{\tilde{\lambda }}_{o}$$: Retardation time
$k$: Permeability of porous medium
$$\overset{\lower0.5em\hbox{$\smash{\scriptscriptstyle\smile}$}}{D}_{nf}$$: Chemical diffusivity of nanofluid
$$\overset{\lower0.5em\hbox{$\smash{\scriptscriptstyle\smile}$}}{K}$$: Permeability of medium
$$\dddot M$$: Magnetic parameter
$$\tilde{R}$$: Radiation parameter
LT: Laplace transform
$$f$$: Base fluid
$$\overset{\lower0.5em\hbox{$\smash{\scriptscriptstyle\frown}$}}{\tilde{\sigma }}_{nf}$$: Electrical conductivity of nanofluid
$$\left( {\overset{\lower0.5em\hbox{$\smash{\scriptscriptstyle\frown}$}}{\beta }_{T} } \right)_{nf}$$: Thermal expansion of nanofluid
$$\overset{\lower0.5em\hbox{$\smash{\scriptscriptstyle\frown}$}}{g}$$: Acceleration due to gravity
$$\left( {\overset{\lower0.5em\hbox{$\smash{\scriptscriptstyle\frown}$}}{\beta }_{C} } \right)_{nf}$$: Volumetric expansion of nanofluid
$\tilde{q}_{r}$: Radiative heat flux in $\tilde{Y}$ direction
$k_{nf}$: Thermal conductivity of nanofluid
$\hat{G}_{T}$: Thermal Grashof number
$\hat{G}_{C}$: Mass Grashof number
$$\Pr$$: Prandtl number
$$Sc$$: Schmidt number
$$nf$$: Nanofluid
$s$: Solid nano-particles
